# Supplementary material for: Deciphering the allosteric regulation of mycobacterial inosine-5′-monophosphate dehydrogenase
Source: Nat Commun. 2024 Aug 6;15:6673. doi: 10.1038/s41467-024-50933-6 (PMC11303537; doi:10.1038/s41467-024-50933-6)
Supplement: Supplementary file 1 — Supplementary Information [file 41467_2024_50933_MOESM1_ESM.pdf]

Deciphering the allosteric regulation of mycobacterial inosine-5′-monophosphate dehydrogenase

Ondřej Bulvas, Zdeněk Knejzlík, Jakub Sýs, Anatolij Filimoněnko, Monika Čížková, Kamila Clarová, Dominik Rejman, Tomáš Kouba* & Iva Pichová*

Supplementary Figures

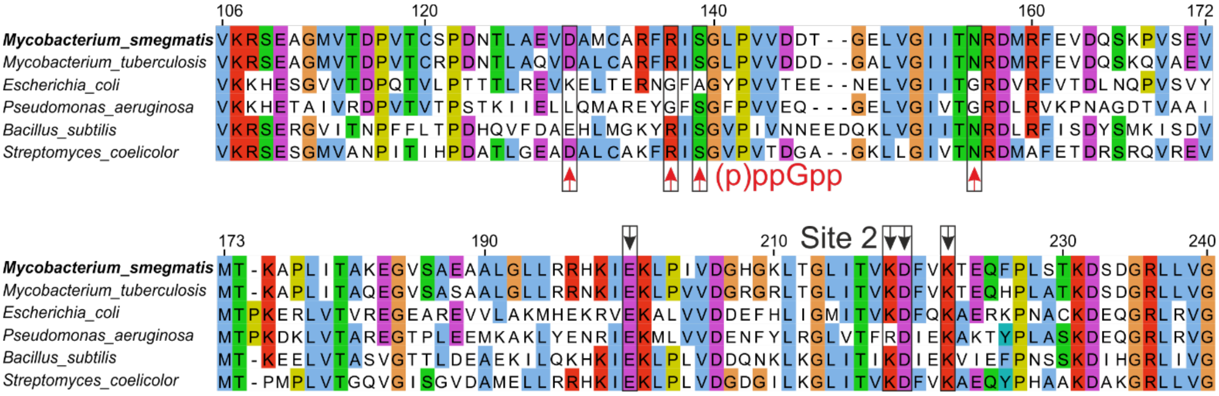

Supplementary Fig. 1 | Conservation of nucleotide binding sites in the CBS domains of bacterial IMPDHs. Multiple sequence alignment of CBS domains for selected bacterial IMPDHs shows the conservation of canonical binding Site 2. The most important residues for nucleotide binding are indicated by red arrows for (p)ppGpp and black arrows for Site 2.

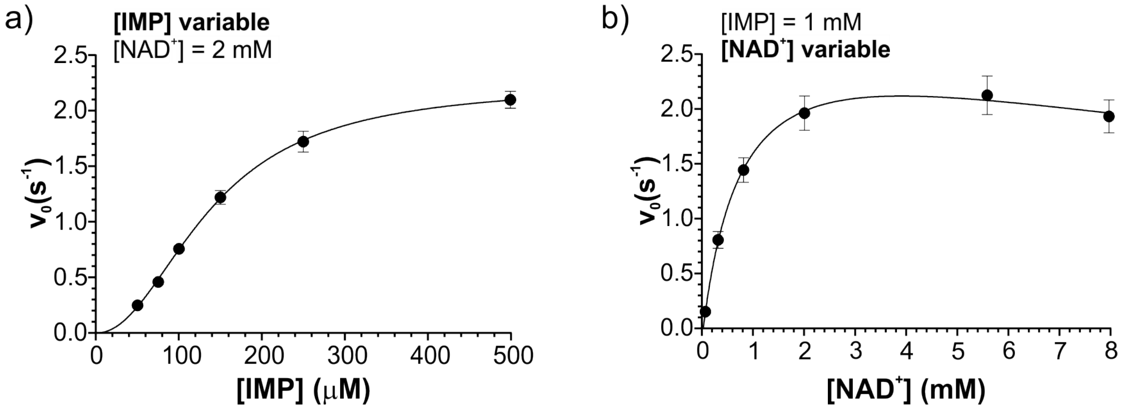

Supplementary Fig. 2 | Plots of the reaction rate of *Msm*IMPDH versus IMP and NAD$^+$ concentrations. a,**b**, IMPDH reaction kinetics indicate Hill dependence and Michaelis–Menten dependence on IMP (**a**) and NAD$^+$ (**b**) concentrations (n = 3), respectively. *Msm*IMPDH was inhibited by NAD$^+$ due to the formation of a dead-end E~XMP intermediate NAD$^+$ complex. Data are presented as mean values with error bars representing the SD.

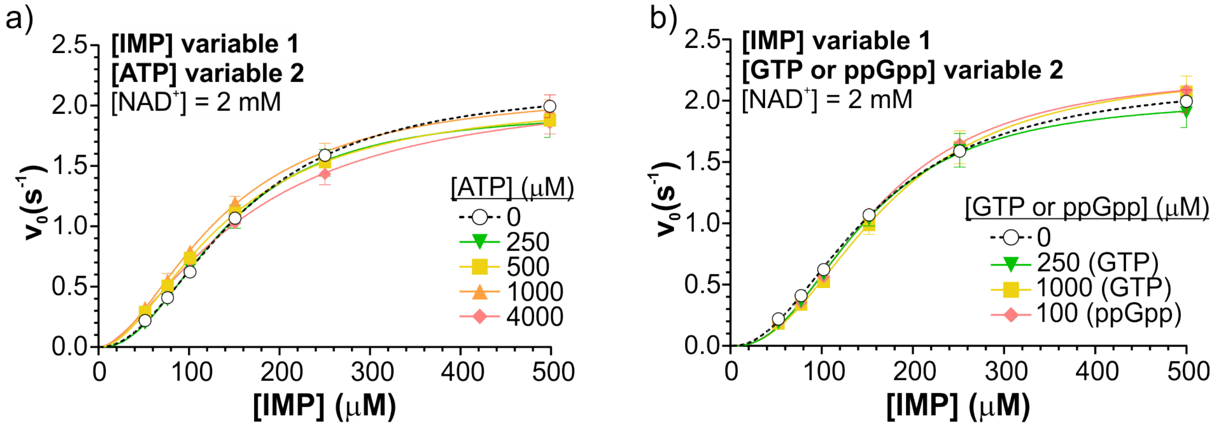

Supplementary Fig. 3 | Plots of the reaction rate of *Msm*IMPDH versus IMP in the presence of different variables. **a**,**b**, Reaction rates versus IMP concentration (n = 3) in the presence of ATP alone (**a**), GTP alone, or ppGpp alone (**b**). The Hill equation was fitted to the initial velocity versus IMP concentration. The black dashed line indicates the control reaction without nucleotides. Individually, ATP, GTP, and ppGpp do not influence *Msm*IMPDH kinetics. Data are presented as mean values with error bars representing the SD.

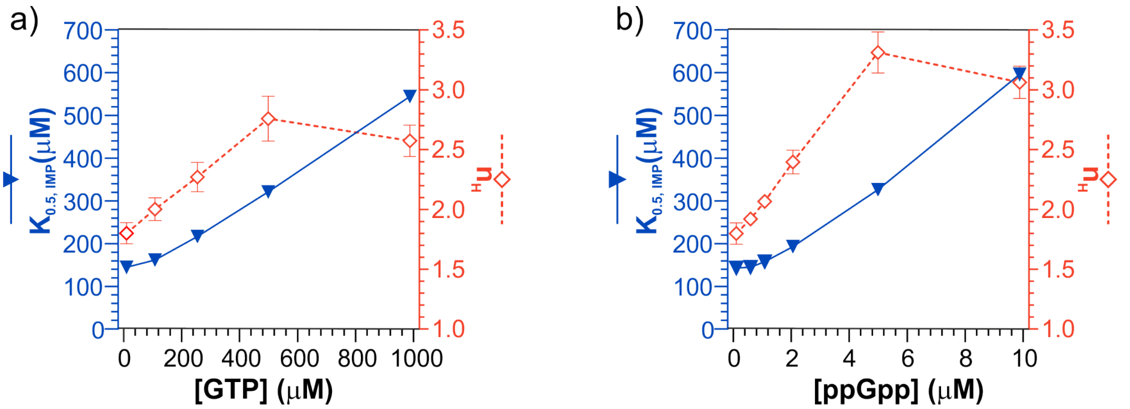

Supplementary Fig. 4 | Plots of Hill equation parameters for GTP- and ppGpp-inhibited reactions of *Msm*IMPDH. **a**,**b**, Hill equation parameters ($K_{0.5,\ IMP}$ and n_H) are plotted as a function of GTP (**a**) or ppGpp (**b**) concentration. Data are presented as mean values with error bars representing the SD.

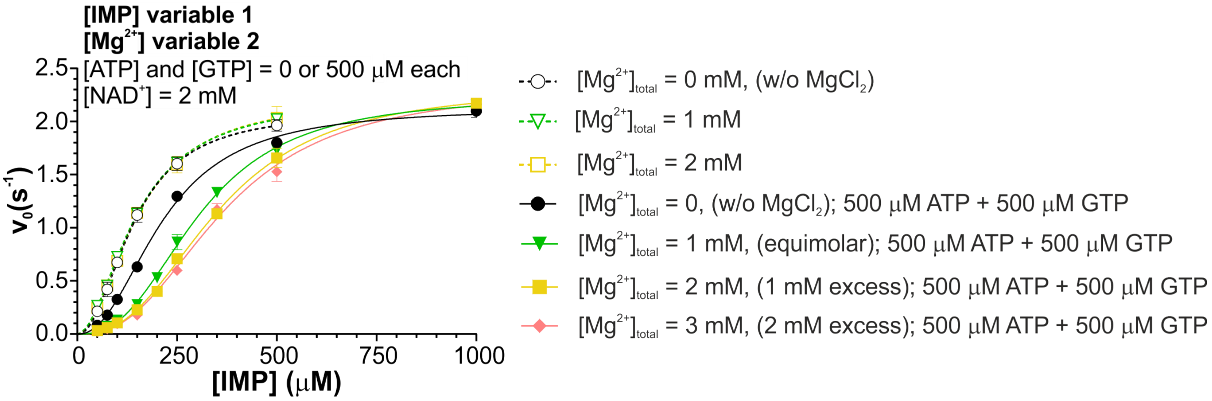

Supplementary Fig. 5 | The effect of the magnesium on the GTP/ATP inhibitory effect to *Msm*IMPDH IMP reaction kinetics. Reaction rates versus IMP concentration (n = 2) fitted with the Hill equation in the presence of Mg^{2+} alone or in the presence of 500 mM ATP and GTP. The reactions without nucleotides are shown by empty symbols and dashed line. Full symbols represent reaction with ATP and GTP. The 1 mM excess of Mg^{2+} ions in respect to GTP and ATP is required for their full inhibitory effect. Data are presented as mean values with error bars representing the SD.

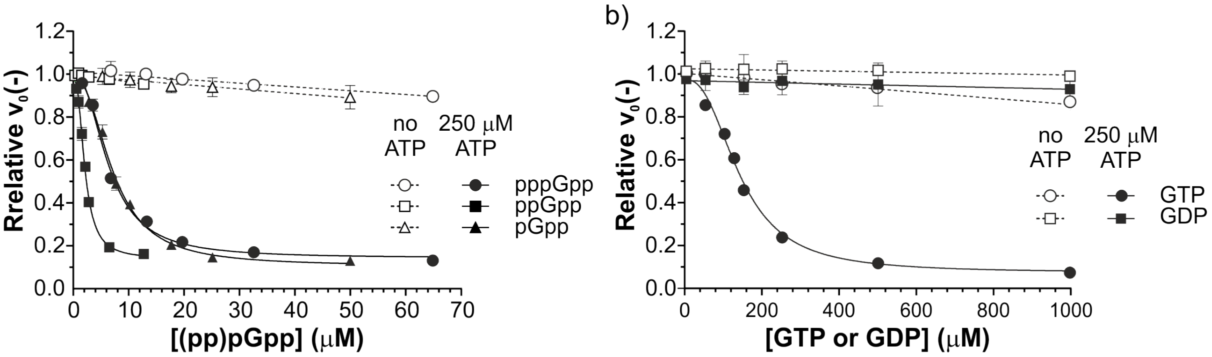

Supplementary Fig. 6 | Plots of *Msm*IMPDH relative velocity versus concentrations of purine effectors. a,**b**, Relative velocities are plotted against pppGpp, ppGpp, or pGpp (**a**) and GTP or GDP (**b**). NAD^+ and IMP substrates were fixed at concentrations of 100 μM and 2 mM (n = 3), respectively. The initial velocity is presented in ratio to the control reaction (y axis) in the presence of varied concentrations of guanine nucleotides (x axis) with and without 250 μM ATP. Data are presented as mean values with error bars representing the SD.

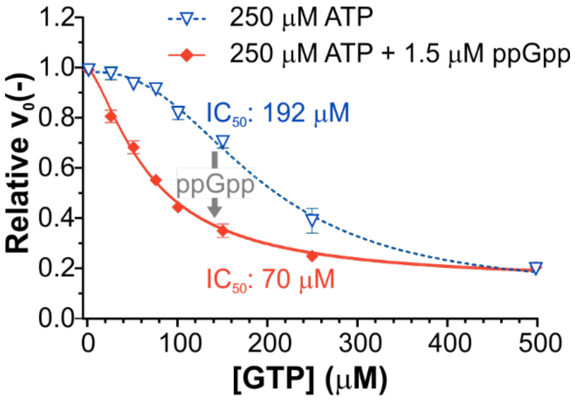

Supplementary Fig. 7 | Effect of ppGpp on *Msm*IMPDH sensitivity to GTP-induced inhibition. The plot of the relative velocity versus GTP concentration (n = 3) alone and with 1.5 μM ppGpp. Initial velocities were measured as a function of GTP concentration under fixed substrate conditions (100 μM IMP and 2 mM NAD^+), along with a constant ATP concentration (250 μM), with and without ppGpp. Relative velocity was calculated as the ratio of the velocities in the presence of the corresponding concentration of GTP to the velocity of the control reaction containing only ATP and ppGpp (n = 3). The presence of 1.5 μM of ppGpp increased the GTP inhibitory effect, shifting the IC_{50} value from 192 ± 6 μM to 70 ± 2 μM. Data are presented as mean values with error bars representing the SD.

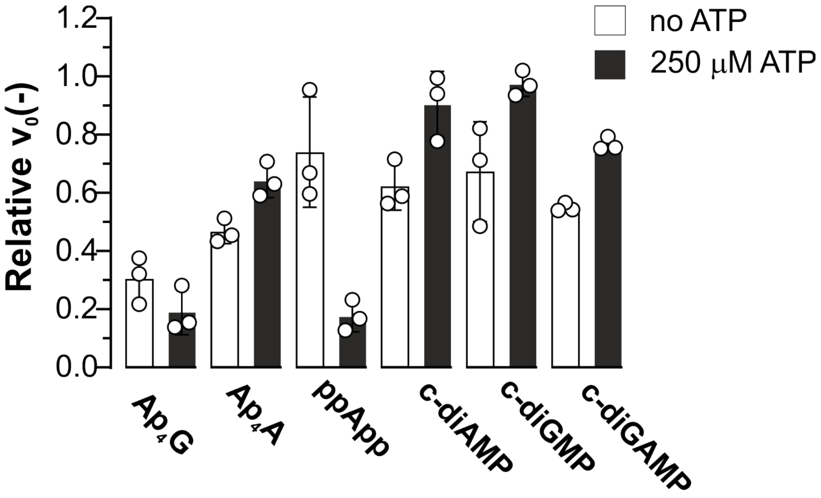

Supplementary Fig. 8 | Effect of selected signalling nucleotides on *Msm*IMPDH activity. The relative velocity of 500 μM nucleotides alone and with 250 μM ATP was calculated as the ratio of the initial velocities of the reaction containing the tested nucleotides to the control reaction (n = 3). Initial velocities were measured at fixed concentrations of 100 μM and 2 mM for the IMP and NAD^+ substrates, respectively. Data are presented as mean values with error bars representing the SD.

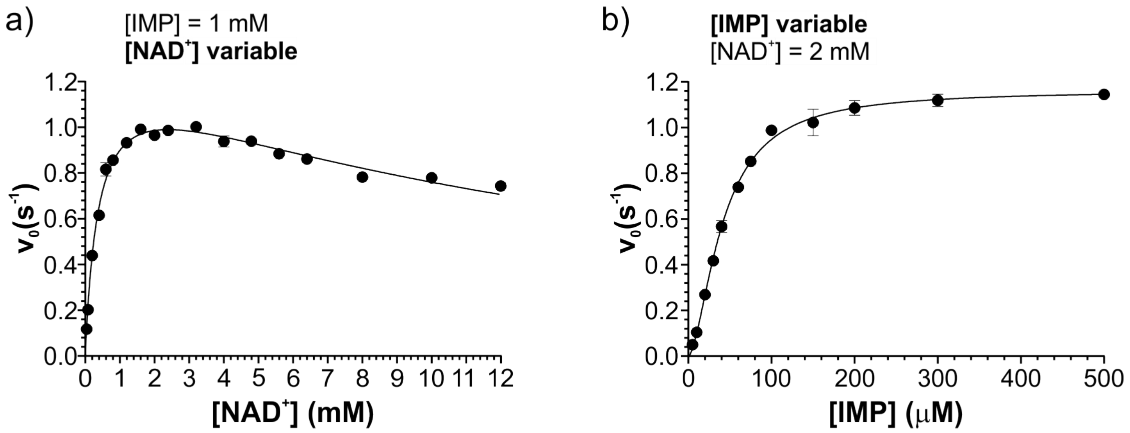

Supplementary Fig. 9 | Plots of the reaction rate of *Mtb*IMPDH versus IMP and NAD$^+$ concentrations. a,**b**, *Mtb*IMPDH reaction kinetics indicate Hill dependence and Michaelis–Menten dependence on IMP (**a**) and NAD$^+$ (**b**) concentrations (n = 2), respectively. *Mtb*IMPDH was inhibited by NAD$^+$ due to the formation of a dead-end E~XMP intermediate NAD$^+$ complex. Data are presented as mean values with error bars representing the SD.

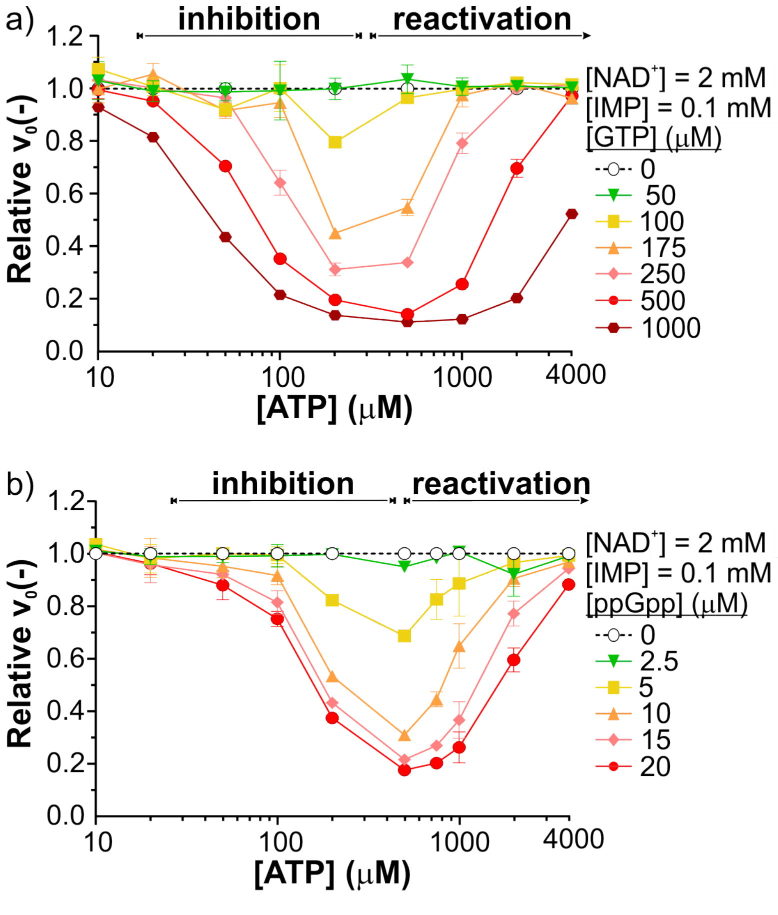

Supplementary Fig. 10 | *Mtb*MPDH is regulated by both GTP and ppGpp in an ATP-dependent manner. a,**b**, Relative velocity is plotted as a function of the ATP concentration at set concentrations of GTP (**a**) and ppGpp (**b**) (n = 2). The substrates NAD$^+$ and IMP were fixed at concentrations of 50 μM and 2 mM, respectively. The relative velocity value was calculated as the ratio of the initial velocity of the reaction in the presence of ATP and GTP or ATP and ppGpp to the control reaction containing only the substrates and corresponding concentration of ATP. Specific ATP concentration ranges above the graphs indicate where inhibition and reactivation occur. Data are presented as mean values with error bars representing the SD.

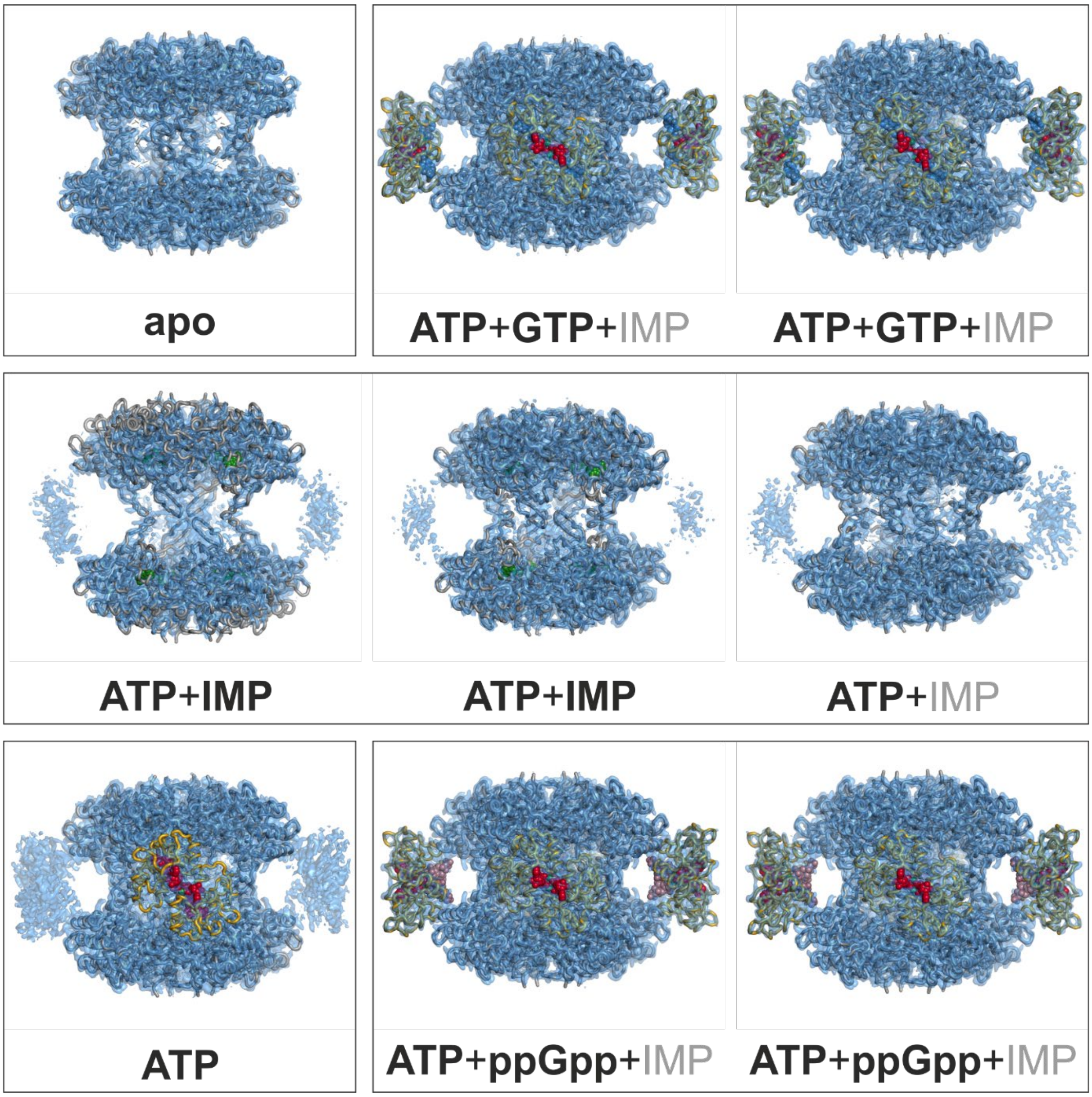

Supplementary Fig. 11 | Overview of *Msm*IMPDH structures obtained by cryo-EM. Experimental cryo-EM maps (in blue) are overlaid with atomic models of *Msm*IMPDH. The black frames group the structures obtained from the same dataset. Bold text indicates which ligands are bound in a given structure, while grey text indicates the ligands that are present under experimental conditions but not in the structure. ATP is shown as red spheres, IMP as purple, GTP as blue, and ppGpp as pink.

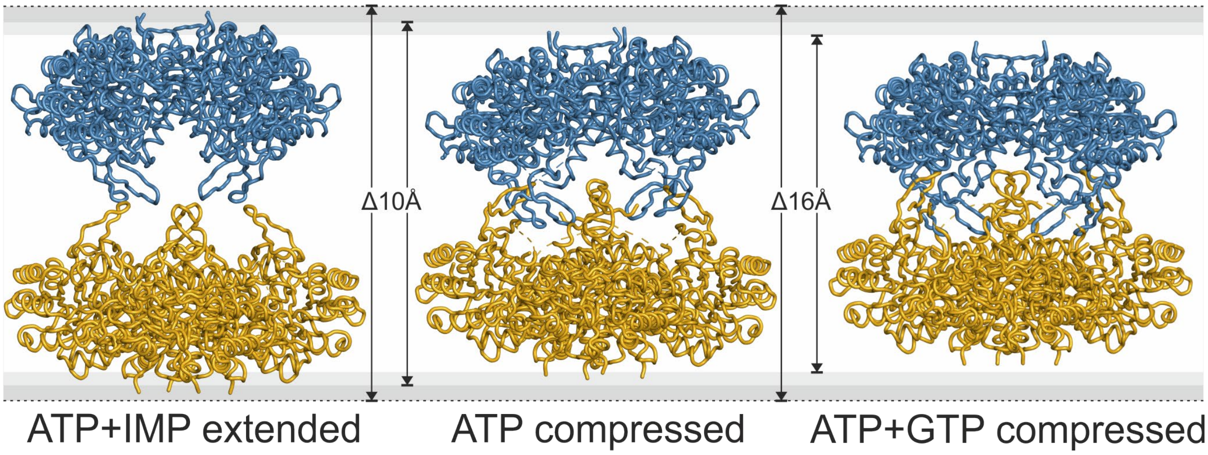

Supplementary Fig. 12 | Conformation dynamics of *Msm*IMPDH octameric assembly. Only catalytic domains of *Msm*IMPDH are visualized. One of the tetramers is depicted in blue, and its opposite tetramer in gold. The tetramers in the extended conformation lose nearly all mutual contacts; the compressed conformation is facilitated by the extensive interactions of the finger and flap loops.

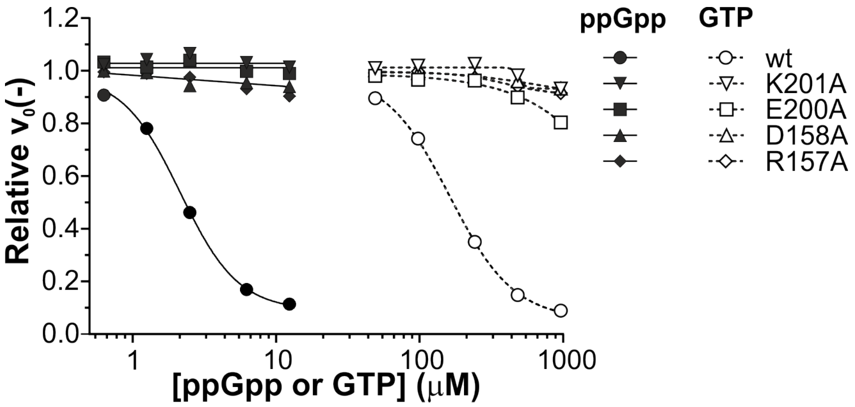

Supplementary Fig. 13 | Sensitivity of ATP site-1 *Msm*IMPDH mutants to GTP- or ppGpp-induced inhibition. NAD^+ and IMP substrates were fixed at concentrations 2 mM and 100 μM, respectively; ATP was fixed at 500 μM. The relative velocity value was calculated as the ratio of the initial velocity of the reaction to the control reaction of the wild-type enzyme (n = 1). Mutations in the residues involved in Site 1 abrogate inhibition by both GTP and ppGpp.

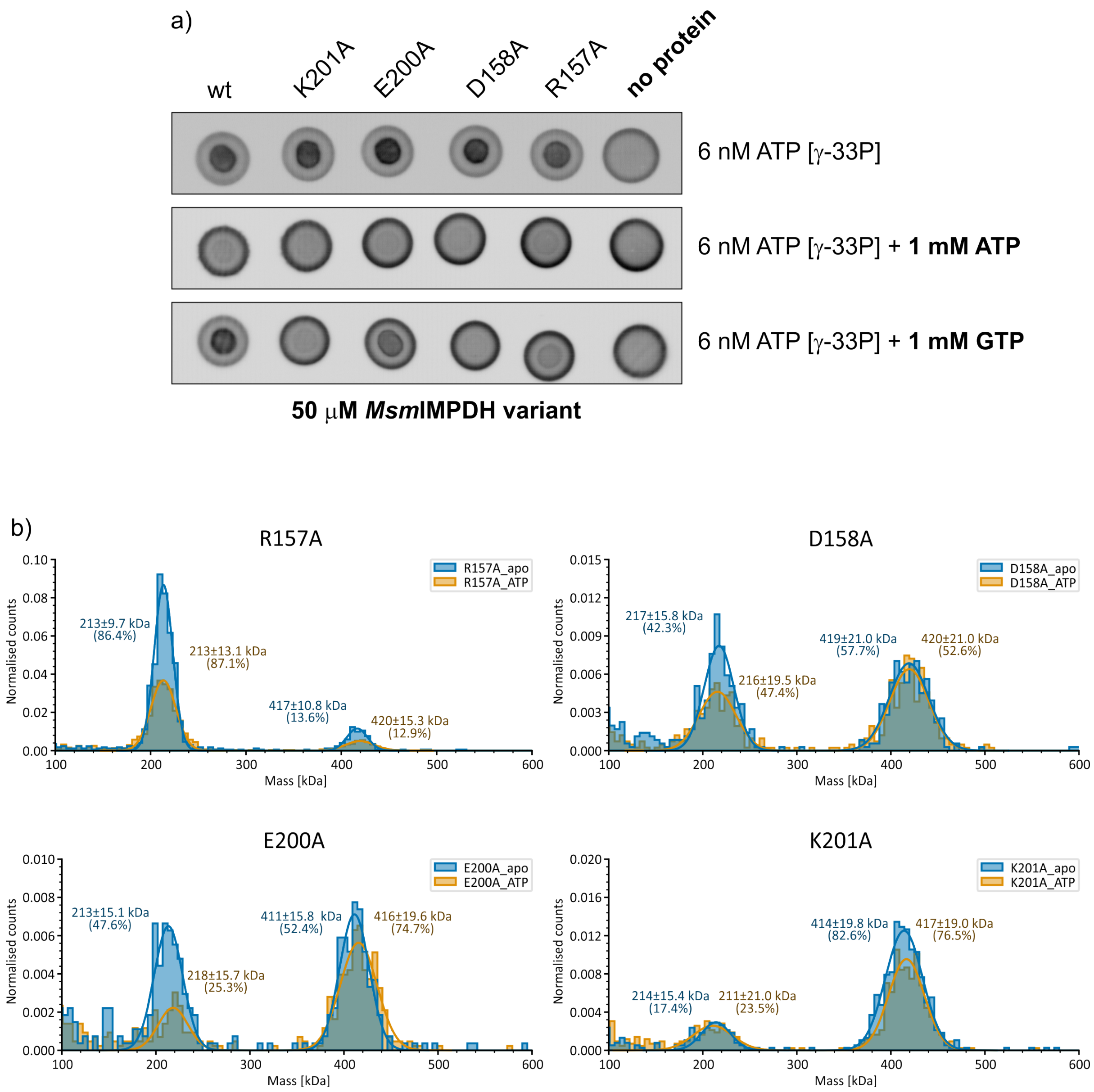

Supplementary Fig. 14 | DRaCALA and mass photometry assays showing ATP binding to WT *Msm*IMPDH and its Site 1 mutants. a, All *Msm*IMPDH variants tested were able to bind radiolabelled ATP [γ-33P] (*top panel*). This binding is competitively suppressed by 1 mM excess of unlabelled ATP (*middle panel*). A significant decrease in the binding of ATP [γ-33P] by Site 1 *Msm*IMPDH mutants occurs in the presence of 1 mM GTP (*bottom panel*). **b**, Mass photometry profiles of 20-nM *Msm*IMPDH mutants in the apo state and in the presence of 5 mM ATP. The observed particle masses of the first and second peaks are consistent with the MsmIMPDH tetramer (213.2 kDa) and the octamer (426.4 kDa).

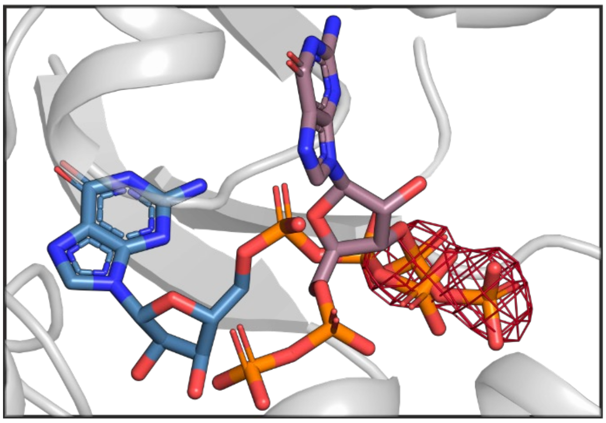

Supplementary Fig. 15 | Binding of GTP at Site 2 and ppGpp at the dedicated ppGpp site is mutually exclusive. The GTP molecule is depicted in blue, and the ppGpp molecule in pink. The red mesh illustrates the potential clash of the δ- and ε-phosphates of ppGpp with the β- and γ-phosphates of GTP.

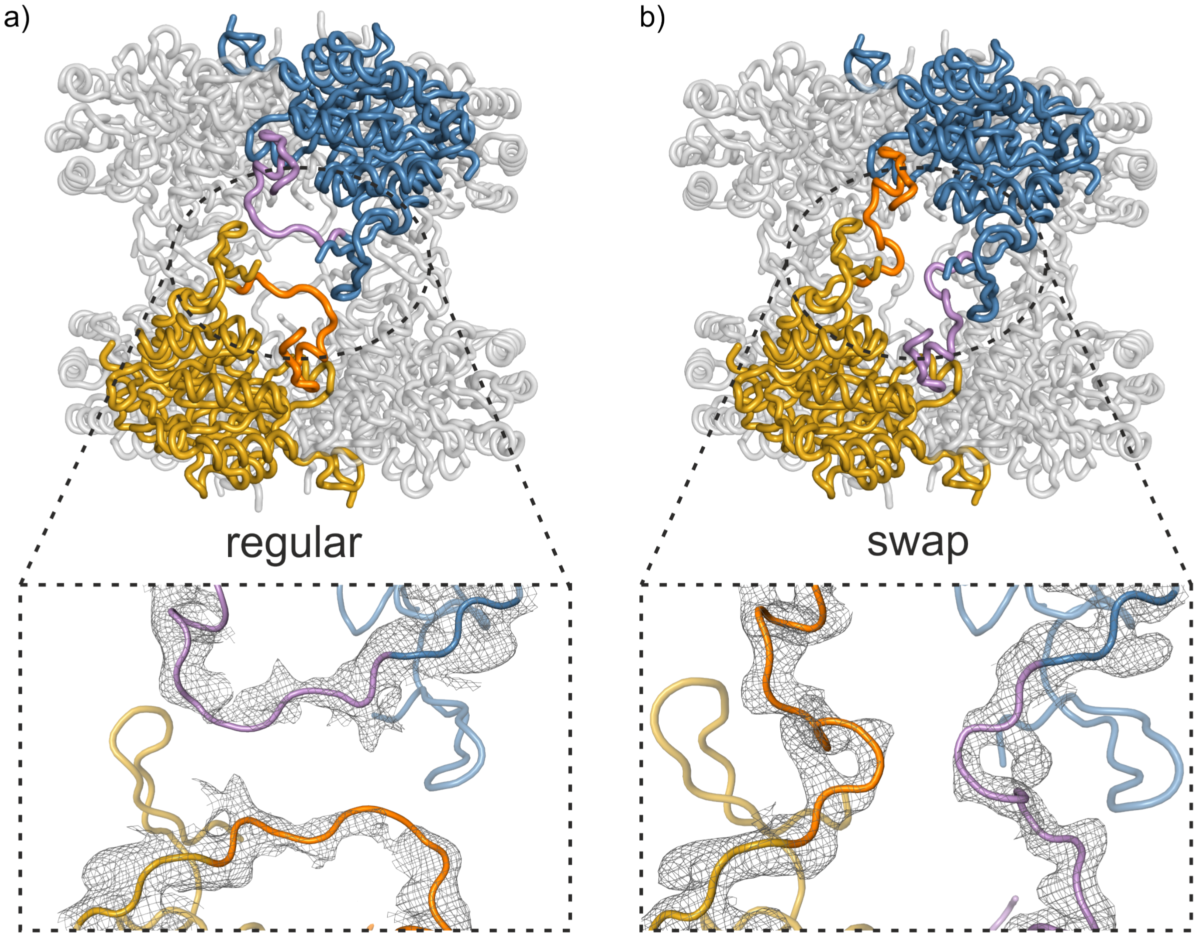

Supplementary Fig. 16 | Swap of flap loops upon "super-compression" of *Msm*IMPDH. a, In the regular arrangement, the flap loops bind to the active site of the resident protomer. Upon tight compression of *Msm*IMPDH, the flap loops reach towards the active site of the opposite protomer. **b**, This movement enables the flap loops to swap from a *cis* to a *trans* configuration; the two opposite *Msm*IMPDH protomers are depicted in gold and blue, respectively. The flap loops are highlighted in orange for the gold protomer and purple for the blue protomer.

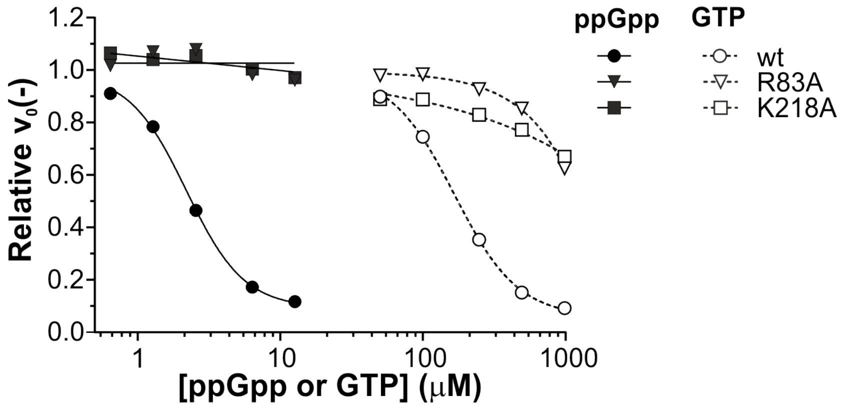

Supplementary Fig. 17 | Sensitivity of mutants in the CBS allosteric site of *Msm*IMPDH to GTP- or ppGpp-induced inhibition. NAD^+ and IMP substrates were fixed at concentrations of 2 mM and 100 μM, respectively; ATP was fixed at 500 μM. The relative velocity value was calculated as the ratio of the initial velocity of the reaction to the control reaction of the wild-type enzyme (n = 1). The mutation of the residues involved in the hinge regions strongly reduced the responsiveness of *Msm*IMPDH to ppGpp and GTP inhibition.

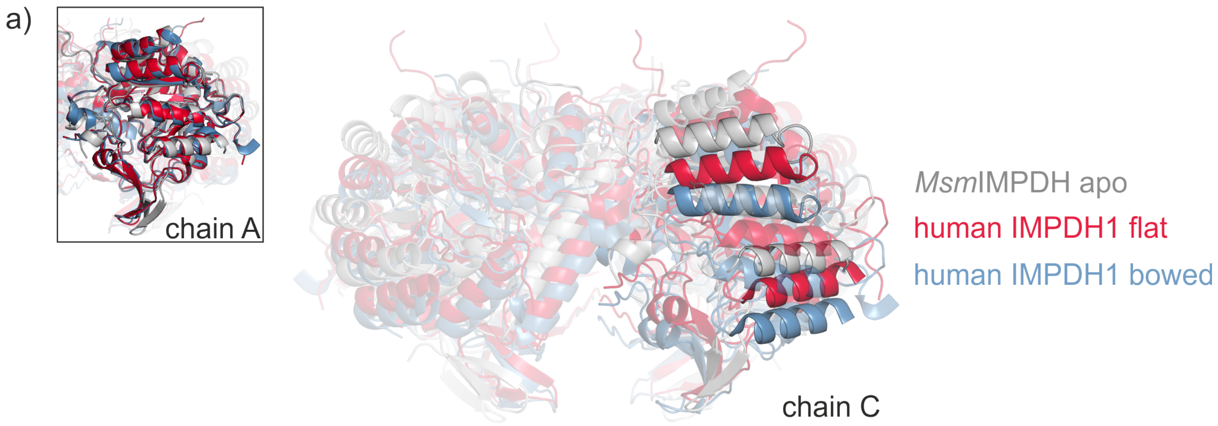

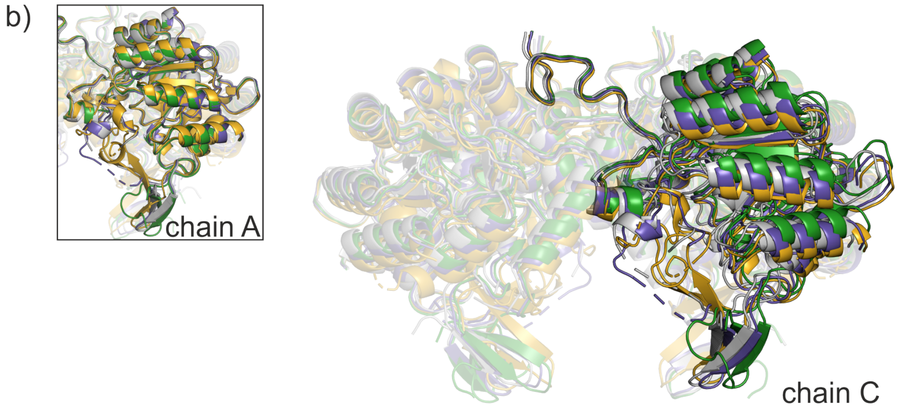

Supplementary Fig. 18 | Flat/bowed-like conformation dynamics of *Msm*IMPDH in comparison with human IMPDH1. a, Comparison of human IMPDH1 in flat (PDB code 7RES; red cartoon) and bowed (PDB code 7RFG; blue cartoon) conformations with *Msm*IMPDH apo form (grey). All structures are aligned with their respective A chains (showed in black box), making the differences most pronounced on the opposite side of the tetramer. The analogous helices of respective protomers are highlighted in bright colours, while the rest of the octamer is shaded. **b**, Comparison of representative *Msm*IMPDH structures showing flat/bowed-like dynamics of the tetrameric assembly. The apo *Msm*IMPDH is depicted in grey, ppGpp-bound super-compressed in green, ATP-bound extended in gold, and ppGpp-bound compressed in purple.

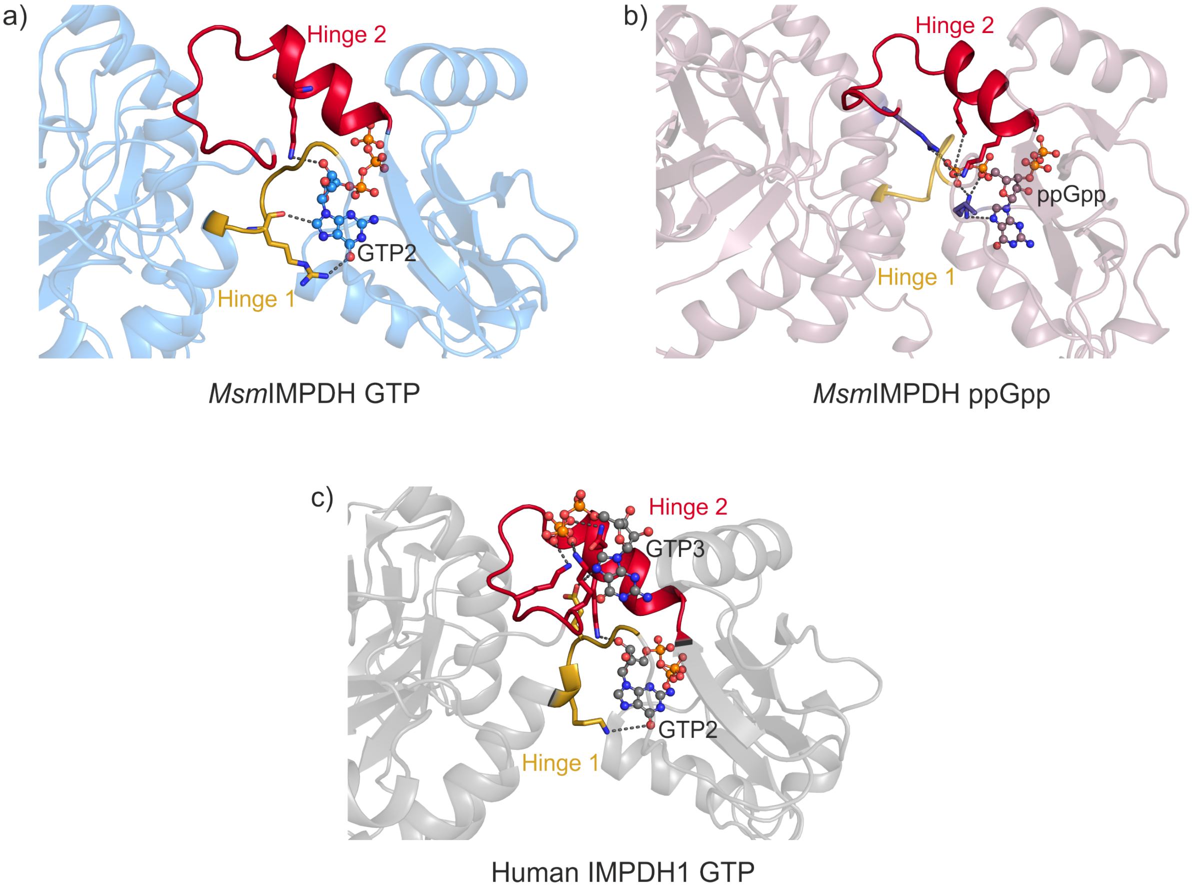

Supplementary Fig. 19 | Comparison of *Msm*IMPDH inhibition mechanism with that of human IMPDH1. a,**b**, Details of the hinge region at the interface between the catalytic and CBS domains of the *Msm*IMPDH compressed octamer with GTP bound at Site 2 (**a**; blue cartoon) or a molecule of ppGpp (**b**; purple cartoon). **c**, Hinge region of human IMPDH 1 (PDB code 7RFG; grey cartoon) with two GTP molecules bound at Site 2 and eukaryotic Site 3. The two hinge regions are highlighted in gold and red, respectively. The GTP and ppGpp ligands are shown as balls and sticks. Dashed lines indicate notable interactions between ligands and residues of the hinge regions.

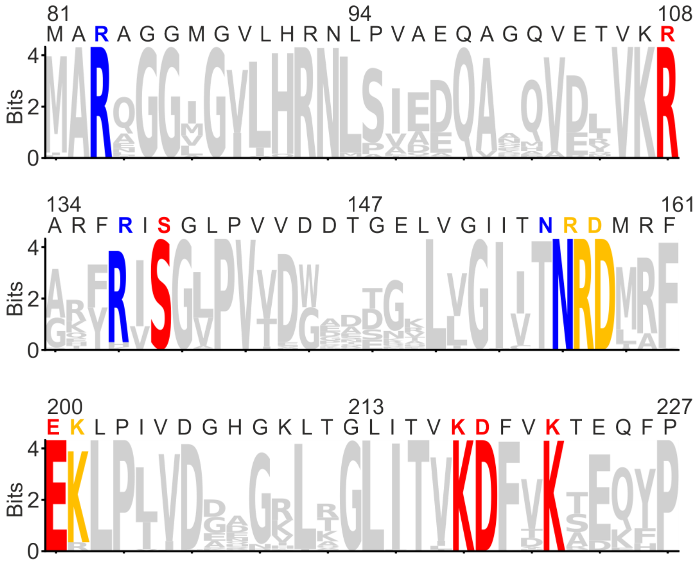

Supplementary Fig. 20 | Conservation of ligand binding sites of actinobacterial IMPDHs. Sequence logo showing the degree of conservation of the CBS-domain binding sites for the IMPDH sequences of all available representatives (2,846 sequences) of the Actinomycetia class. The coloured amino acid residues directly participate in ligand binding, where yellow is ATP binding Site 1, red is Site 2, and blue is the (p)ppGpp site. The *x* axis represents the position of the amino acid in the consensus sequence, and the *y* axis represents the information content in bits.

Supplementary Tables

Supplementary Table 1 | *Msm*IMPDH kinetic parameters (*n* = 3)

Parameter	Fixed [NAD^+], 2 mM variable [IMP]	Fixed [IMP], 1 mM variable [NAD^+]
$k_{cat,\ app}$ (s^{-1})	2.24 ± 0.05	3.0 ± 0.3
$K_{0.5,\ IMP}$ (μM)	139 ± 4	
N_h	2.1 ± 0.1	
$K_{m,\ NAD^+}$ (mM)		0.80 ± 0.17
K_i (mM)		19 ± 7

Supplementary Table 2 | *Msm*IMPDH kinetic parameters of various ligands (*n* = 2–3)

[ATP] (mM)	[GTP] (mM)	[ppGpp] (μM)	[$MgCl_2$] (mM)	$k_{cat,\ app}$ (s^{-1})	$K_{0.5,\ IMP}$ (μM)	n_H
				2.24 ± 0.05	139 ± 4	2.07 ± 0.09
			1	2.18 ± 0.05	154 ± 5	2.03 ± 0.09
0.25			1.25	1.97 ± 0.07	139 ± 7	2.18 ± 0.17
0.5			1.5	2.07 ± 0.02	139 ± 2	1.79 ± 0.03
1			2	2.16 ± 0.07	135 ± 7	1.77 ± 0.11
4			5	2.17 ± 0.09	161 ± 12	1.56 ± 0.1
	0.25		1.25	2.03 ± 0.07	146 ± 7	2.31 ± 0.17
	1		2	2.27 ± 0.09	168 ± 9	2.20 ± 0.16
		100	1.1	2.25 ± 0.04	158 ± 4	2.24 ± 0.07
0.5	0.1		1.6	2.06 ± 0.05	156 ± 6	2.00 ± 0.10

0.5	0.25		1.75	2.03 ± 0.04	213 ± 6	2.27 ± 0.13
0.5	0.5		2	2.03 ± 0.05	320 ± 9	2.77 ± 0.19
0.5	1		2.5	2.01 ± 0.04	549 ± 12	2.58 ± 0.14
0.5		0.5	1.5	2.11 ± 0.02	141 ± 3	1.91 ± 0.05
0.5		1	1.5	2.12 ± 0.03	154 ± 3	2.06 ± 0.05
0.5		2	1.5	2.10 ± 0.03	190 ± 4	2.39 ± 0.10
0.5		5	1.5	2.12 ± 0.04	325 ± 6	3.32 ± 0.18
0.5		10	1.5	2.45 ± 0.05	598 ± 11	3.07 ± 0.14
			1	2.07 ± 0.03	163 ± 5	1.15 ± 0.03
			1	2.16 ± 0.06	252 ± 17	1.06 ± 0.05
			1	2.22 ± 0.09	369 ± 36	1.10 ± 0.06
0.5	0.5		2	2.18 ± 0.04	332 ± 15	1.26 ± 0.04
0.5		5	1.5	2.22 ± 0.04	367 ± 17	1.12 ± 0.03

Supplementary Table 3 | IC$_{50}$ values of guanine nucleotides at 250 µM ATP and fixed substrate concentrations (100 µM IMP, 2 mM NAD$^+$) (*n* = 3)

Nucleotide	IC$_{50}$ at 250 µM ATP (µM)
pppGpp	6.2 ± 0.1
ppGpp	1.8 ± 0.1
pGpp	6.9 ± 0.2
GTP	140 ± 3
GDP	No inhibition

Supplementary Table 4 | *Mtb*IMPDH kinetic parameters (*n* = 2)

Parameter	Fixed [NAD^+], 2 mM variable [IMP]	Fixed [IMP], 1 mM variable [NAD^+]
$k_{cat, app}$ (s^{-1})	1.16 ± 0.01	1.32 ± 0.03
$K_{0.5, IMP}$ (μM)	41 ± 1	
n_h	1.68 ± 0.06	
K_{m, NAD^+} (mM)		0.40 ± 0.03
K_i (mM)		14 ± 1

Supplementary Table 5 | Cryo-EM data collection, refinement, and validation statistics

	Gua2B apo	Gua2B-ATP	Gua2B-ATP+IMP extended	Gua2B-ATP+IMP compressed	Gua2B-ATP+IMP intermediate	Gua2B-GTP+ATP compressed	Gua2B-GTP+ATP super-compressed	Gua2B-ppGpp+ATP compressed	Gua2B-ppGpp+ATP super-compressed
**PDB ID**	8PW3	8Q65	8QQV	8QQW	8QQX	8QQQ	8QQP	8QQT	8QQR
**EMDB ID**	EMD–17988	EMD–18184	EMD–18606	EMD–18607	EMD–18608	EMD–18601	EMD–18600	EMD–18604	EMD–18602
**Data collection and processing**									
Microscope	Titan Krios	Titan Krios	Titan Krios	Titan Krios	Titan Krios	Titan Krios	Titan Krios	Titan Krios	Titan Krios
Voltage (kV)	300	300	300	300	300	300	300	300	300
Camera	Gatan K2	Gatan K2	Gatan K3	Gatan K3	Gatan K3	Gatan K3	Gatan K3	Gatan K3	Gatan K3
Magnification (x)	165 000	165 000	165 000	165 000	165 000	165 000	165 000	165 000	165 000
Nominal defocus range (negative μm)	0.6–3.2	0.4–2.9	0.5–2.6	0.5–2.6	0.5–2.6	0.4–2.8	0.4–2.8	0.3–2.3	0.3–2.3
Exposure time (s)	5	5	2	2	2	2	2	2	2
Electron exposure (e–/Å2)	55	55	43	43	43	44	44	42	42
Number of frames collected	40	40	40	40	40	40	40	40	40
Number of frames processed	24	24	22	22	22	22	22	22	22
Pixel size (Å)	0.818	0.818	0.8336	0.8336	0.8336	0.8336	0.8336	0.8336	0.8336
Micrographs	8 664	6 484	11 232	11 232	11 232	40 440	40 440	37 330	37 330
Total particle images	5 882 510	2 754 934	7 395 061	7 395 061	7 395 061	26 489 203	26 489 203	15 670 460	15 670 460
**Refinement**									
Particles per class	550 995	317 151	218 003	69 863	219 594	732 918	715 534	428 872	348 496
Map resolution (Å),0.143 FSC	2.507	2.54	2.99	3.27	3.01	2.43	2.35	2.76	2.73
Map sharpening B factor (Å2)	–54.4	–61.99	–84.89	–78.57	–91.12	–67.93	–58.13	–78.79	–75.85
Map versus model cross-correlation	0.82	0.88	0.87	0.88	0.87	0.89	0.89	0.9	0.91

Continued on the next page

Model composition									
Non-hydrogen atoms	19 800	21 366	21 087	19 552	20 424	27 301	27 622	27 175	27 632
Protein residues	2 752	2 946	2 847	2 728	2 792	3 656	3 712	3 655	3 712
Chains	8	8	9	8	8	8	8	8	8
Water	0	0	29	0	0	149	110	0	0
Ligands	0	ATP:4 MG:4	IMP:8	0	IMP:8	ATP:8 GTP:8 MG:16	ATP:8 GTP:8 MG:16	ATP:8 G4P:8 MG:16	ATP:8 G4P:8 MG:16
B factors (Å2) *min/max/mean*									
Protein	0.10/97.33/36.99	0.09/128.13/38.33	16.70/165.87/70.33	0.06/125.83/50.20	5.92/124.87/50.20	0.90/138.87/47.23	1.54/128.53/48.42	2.44/130.68/50.05	1.92/118.55/46.71
Ligand	---	102.41/129.96/119.95	62.72/82.21/72.69	---	48.51/81.53/68.50	35.18/82.97/60.30	56.36/93.61/75.98	34.55/61.15/50.77	28.95/73.70/52.67
Water	---	---	22.95/76.48/48.71	---	---	28.03/56.02/40.31	21.84/87.50/41.65	---	---
R.m.s. deviations									
Bond lengths (Å)	0.003	0.003	0.01	0.002	0.002	0.003	0.003	0.002	0.003
Bond angles (°)	0.552	0.495	0.762	0.438	0.502	0.529	0.525	0.464	0.519
Validation									
MolProbity score	1.1	1.14	1.08	1.13	1.1	0.93	1.07	1.11	1.03
All-atom clashscore	3.1	3.54	2.92	3.36	2.74	1.75	2.65	3.24	2.46
Rotamer outliers (%)	0.39	0.77	0.79	0	0.67	0.5	0.25	0.75	0.39
Ramachandran plot									
Favoured (%)	98.36	98.23	98.35	98.91	97.84	98.09	97.93	98.34	99.24
Allowed (%)	1.64	1.77	1.65	1.09	2.16	1.91	2.07	1.66	0.76
Outliers (%)	0	0	0	0	0	0	0	0	0

Supplementary Table 6 | Kinetic parameters of *Msm*IMPDH mutants (*n* = 2)

*Msm*IMPDH variant	Region	$k_{cat, app}$ (s^{-1})	$K_{0.5, IMP}$ (μM)	n_H
wt		2.18 ± 0.04	127 ± 3	1.99 ± 0.07
K222A	hinge	2.29 ± 0.10	197 ± 12	2.06 ± 0.14
R108A	hinge	2.21 ± 0.04	92 ± 3	1.54 ± 0.07
R83A	hinge	2.25 ± 0.06	109 ± 5	1.52 ± 0.09
K218A	hinge	2.13 ± 0.06	226 ± 11	1.61 ± 0.08
K201A	Site 1	2.20 ± 0.05	88 ± 3	1.59 ± 0.10
E200A	Site 1	2.20 ± 0.04	112 ± 4	1.74 ± 0.08
D158A	Site 1	2.09 ± 0.03	82 ± 2	1.59 ± 0.07
R157A	Site 1	2.18 ± 0.08	91 ± 6	1.55 ± 0.15

Supplementary Table 7 | List of plasmids used in this study

**Plasmid**	**Source**
pRSF-HisTEV	Laboratory stock
pRSF-HisTEV-MsmGuaB2	This study
pRSF-HisTEV-MsmGuaB2-K201A	This study
pRSF-HisTEV-MsmGuaB2-E200A	This study
pRSF-HisTEV-MsmGuaB2-D158A	This study
pRSF-HisTEV-MsmGuaB2-R157A	This study
pRSF-HisTEV-MsmGuaB2-K222A	This study
pRSF-HisTEV-MsmGuaB2-R108A	This study
pRSF-HisTEV-MsmGuaB2-R83A	This study
pRSF-HisTEV-MsmGuaB2-K218A	This study
pET24d-HS	Laboratory stock
pET24d-HS-MtbGuaB2	This study

Supplementary Table 8 | List of oligonucleotides used in this study

Primer number	Description	Sequence
#1	MsmGuaB2 WT for	CAGGATCCGAATTCGGAAAACCTGTATTTTCAGGGCTCGATCGCTGAAAGCAGCGTTCCCATCGCCG
#2	MsmGuaB2 WT rev	CCTGCAGGCGCGCCGTCAGCGGGTGTAGTAGTTGGGTG
#3	MsmGuaB2 K201A for	CGCAGGCACAAGATCGAGGCACTGCCGATCGTG
#4	MsmGuaB2 K201A rev	GATCTTGTGCCTGCGCAGCAG
#5	MsmGuaB2 E200A for	CTGCGCAGGCACAAGATCGCAAAGCTGCCGATC
#6	MsmGuaB2 E200A rev	GATCTTGTGCCTGCGCAGCAG
#7	MsmGuaB2 D158A for	GGCATCATCACCAACCGCGCAATGCGCTTCGAG
#8	MsmGuaB2 D158A rev	GTTGGTGATGATGCCCACCAA
#9	MsmGuaB2 R157A for	GTGGGCATCATCACCAACGCAGACATGCGCTTC
#10	MsmGuaB2 R157A rev	GTTGGTGATGATGCCCACCAA
#11	MsmGuaB2 K222A for	ATCACCGTCAAGGACTTCGTCGCAACCGAGCAGTTC
#12	MsmGuaB2 K222A rev	GACGAAGTCCTTGACGGTGAT
#13	MsmGuaB2 R108A for	GGCCAGGTCGAGACGGTCAAAGCCTCCGAGGCGGGCATG
#14	MsmGuaB2 R108A rev	TTTGACCGTCTCGACCTGGCC
#15	MsmGuaB2 R83A for	CGCATGGCCATCGCGATGGCGGCAGCGGGCGGCATG
#16	MsmGuaB2 R83A rev	CGCCATCGCGATGGCCATGCG
#17	MsmGuaB2 K218A for	ACGGGCCTCATCACCGTCGCAGACTTCGTCAAG
#18	MsmGuaB2 K218A rev	GACGGTGATGAGGCCCGTGAG
#19	MtbGuaB2 WT for	GAACAGATTGGTGGGTCCCGTGGCA
#20	MtbGuaB2 WT rev	ATTCGGATCCGGTCTTTAGCGCGCGTAGTAGTTGGG
#21	pET24d-HS for	AGACCGGATCCGAATTCGAG
#22	pET24d-HS rev	CCCACCAATCTGTTGCGAT

Supplementary Table 9 | HDX-MS data summary table

Data Set	apo (IMPDH)	ATP	ATP+IMP	ATP+IMP+GTP
HDX reaction details	50mM Tris, pD_{read} = 8, 4 °C, protein:D_2O ratio 1:4	50mM Tris, pD_{read} = 8, 4 °C, protein:D_2O ratio 1:4	50mM Tris, pD_{read} = 8, 4 °C, protein:D_2O ratio 1:4	50mM Tris, pD_{read} = 8, 4 °C, protein:D_2O ratio 1:4
HDX time course (s)	2, 5, 10, 20, 120	2, 5, 10, 20, 120	2, 5, 10, 20, 120	2, 5, 10, 20, 120
HDX control samples	Reference samples of *Msm*IMPDH (nondeuterated) were prepared as follows: 10 µl of 40µM *Msm*IMPDH was equilibrated using H2O-based equilibration buffer (50mM Tris, pH 8), and quenched by an addition of equal volume of quench buffer (0.1mM Phosphate, pH 2.51). The final amount of *Msm*IMPDH subjected to the mass-spectrometric analysis was 100 pmol per each sample.			
# of Peptides	221	221	172	172
Sequence coverage	97%	97%	94%	94%
Average peptide length / Redundancy	11.47/5.08	11.47/5.08	11.34/4.05	11.34/4.05
Replicates (biological or technical)	3 (technical)	3 (technical)	3 (technical)	3 (technical)
Repeatability	0.048 (average standard deviation)	0.069 (average standard deviation)	0.047 (average standard deviation)	0.059 (average standard deviation)
Significant differences in HDX (delta HDX > X D)	0.419 D (98% CI)		0.402 D (98% CI)	

Supplementary Table 10 | SAXS data collection, processing, and validation parameters

Name of the sample	*Msm* IMPDH apo	*Msm* IMPDH 10 mM IMP	*Msm* IMPDH 10 mM IMP+ATP	*Msm* IMPDH 10 mM ATP	*Msm* IMPDH 10 mM GTP
Source organism of the protein	*Micobacterium smegmatis*				
Source (catalogue No. or reference)	*E. coli* BL21 LOBSTR				
M_W (theoretical) [Da]	53300				
Concentration of the sample	3 mg/ml				
Buffer	50mM HEPES pH 7.5, 200mM KCl, 2mM MgCl2, 0.5M TCEP				
X-ray/neutron source	Anton Paar MetalJet				
Wavelength [nm]	0.134				
Beam size at sample [mm]	0.89	0.89	0.93	0.93	0.89
Detector	Eiger R 1M				
Beamstop size [mm]	2				
Sample to detector distance [m]	0.797800003				
Exposure time [s]	60x31s	60x31s	60x31s	40x31s	60x31s
q-range [nm]	0.062 - 4.283	0.062 - 4.283	0.066 - 4.281	0.066 - 4.281	0.062 - 4.283
Temperature [K]	293				
SASBdb ID	SASDUM5	SASDUN5	SASDUQ5	SASDUP5	SASDUR5
Data q-range	0.083 - 3.997	0.083 - 3.997	0.083 - 3.997	0.083 - 3.997	0.083 - 3.997
Guinier analysis					
R_g	5.31 ± 0.04	4.98 ± 0.05	5.07 ± 0.04	5.05 ± 0.05	4.98 ± 0.03
I(0)	0.72 ± 0.004	0.243 ± 0.002	0.404 ± 0.002	0.405 ± 0.003	0.373 ± 0.002
Point index, of the Guinier region	2 - 28	8 - 31	4 - 30	1 - 29	1 - 31
q-range [nm]	0.089 - 0.241	0.124 - 0.258	0.100 - 0.253	0.083 - 0.247	0.083 - 0.258
Fidelity	0.825	0.018	0.319	0.323	0.168
Data quality	0.718	0.553	0.518	0.393	0.652
Aggregation index	-0.00135922	0.025763	0.000365024	0	0
Shannon analysis					
Optimal Shannon channels	19	15	10	9	14
Optimal q_{max}	3.307	2.957	2.135	1.648	2.946
Molecular weight estimates					
MW from Porod Volume					
Vp	793355	466026	669433	691988	713917
M_W [Da]	495847	291266	418396	432493	446198
M_W from Porod Invariant					
M_W [Da]	488985	282974	427317	445661	432766

Continued on the next page

M_W from Volume of Correlation					
Vc	1686.15	1282.27	1647.64	1656.86	1585.47
M_W [Da]	435080	268162	434596	441942	410276
M_W from Apparent Volume					
Q	0.0000252	0.0000386	0.0000247	0.0000218	0.0000258
V_{app}	782857	511767	799316	904369	764248
M_W [Da]	438772	285118	448100	507644	428224
M_W from Size and Shape					
M_W [Da]	386722	249002	409702	358904	364695
M_W from Bayesian inference					
M_W [Da]	392300	318450	392300	318450	318450
M_W_score	0.757428	0.923098	0.773441	0.91714	0.922895
Analysis using indirect fourier transform (GNOM)					
R_g (from IFT)	5.6 ± 0.1	5.2±0.08	4.96 ± 0.01	4.93 ± 0.01	4.86 ± 0.01
I(0) (from IFT)	0.732 ± 0.006	0.2464 ±0.002	0.401±0.001	0.402 ± 0.001	0.37 ± 0.001
q-range [nm]	0.0888 - 1.5047	0.1239 - 1.6041	0.1005 - 1.5749	0.0829 - 1.5807	0.0829 - 1.6041
Real space range	0.0 - 24.5	0.0 - 21.5	0.0 - 13.2	0.0 - 14.78	0.0 - 14.4
Fit quality	0.7228	0.7873	0.9866	0.9761	0.9844
Data/atomic model agreement (Crysol)					
M_W (from PDB)	426000	N/A	N/A	426000	426000
q-range [nm]	0.08 - 1.99	N/A	N/A	0.08 - 1.99	0.08 - 1.99
R_g (theoretical)	5.02	N/A	N/A	5.04	5.00
chi^2	6.895	N/A	N/A	2.433	4.372
Results of ab-initio shape determination (DAMMIF)					
Particle symmetry	P1	N/A	N/A	P1	P1
D_{max} (input)	24.5	N/A	N/A	14.8	14.4
R_g (input)	5.58	N/A	N/A	4.93	4.86
q-range	0.0 - 1.5	N/A	N/A	0.0 - 1.58	0.0 - 1.6
M_W estimate	502000.0 ± 2000.0	N/A	N/A	393000.0 ± 1000.0	375000.0 ± 1000.0
Particle R_g	5.5855 ± 0.0004	N/A	N/A	4.9291 ± 0.0002	4.8603 ± 0.0002
Particle D_{max}	25.0 ± 1.0	N/A	N/A	16.2 ± 0.3	15.4 ± 0.2
Chi^2	1.064 ± 0.009	N/A	N/A	0.976 ± 0.0009	3.14 ± 0.01
damsel					
Number of models on input	32	N/A	N/A	32	32
No. of excluded models	1	N/A	N/A	2	1

Continued on the next page

Results of *ab-initio* shape determination (DAMMIN)					
Number of models	8				
Particle symmetry	P1	N/A	N/A	P1	P1
D_{max} (input)	24.50	N/A	N/A	14.78	14.40
R_g (input)	5.585	N/A	N/A	4.929	4.86
q-range	0.0 - 1.505	N/A	N/A	0.0 - 1.581	0.0 - 1.604
Particle R_g	5.541 ± 0.005	N/A	N/A	4.936 ± 0.002	4.868 ± 0.001
Particle D_{max}	21.2 ± 0.1	N/A	N/A	15.7 ± 0.2	15.0 ± 0.2
Chi^2	1.0283 ± 0.0009	N/A	N/A	1.0376 ± 0.0009	1.111
Mixture analysis (OLIGOMER)					
Real oligomer weights [Da]					
tetramer; octamer	N/A	200000; 400000	N/A	N/A	N/A
compressed; extended	N/A	N/A	400000; 400000	N/A	N/A
Oligomer radii of gyration					
tetramer; octamer	N/A	5.08 ; 4.54	N/A	N/A	N/A
compressed; extended	N/A	N/A	5.01; 5.20	N/A	N/A
Range of Scattering angle:	N/A	0.01-0.20	0.01-0.20	N/A	N/A
Chi^2	N/A	2.21	2.27	N/A	N/A
<MW>	N/A	251151	400000	N/A	N/A
<Rg>	N/A	4.76	5.08	N/A	N/A
Volume fractions					
tetramer; octamer	N/A	0.256±0.003; 0.744±0.004	N/A	N/A	N/A
compressed; extended	N/A	N/A	0.618±0.011; 0.382±0.012	N/A	N/A

Supplementary cryo-EM data

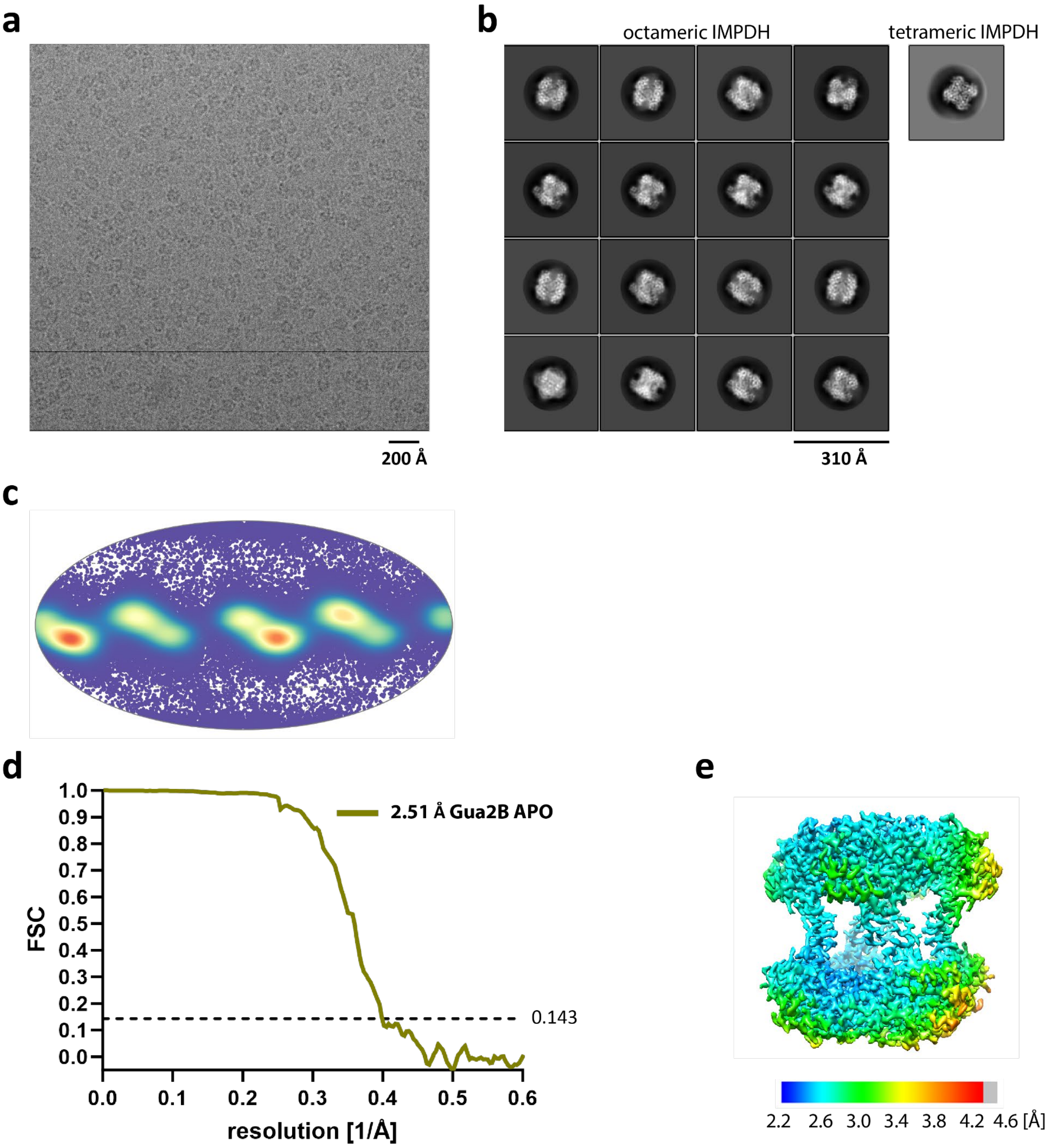

Figure EM-1: Cryo-EM of the *Msm*IMPDH APO form complex

a, Micrograph of the *Msm*IMPDH APO form complexes in free standing ice after MotionCor2[1] correction at defocus of ~2.5 µm. **b**, 2D-class averages of the *Msm*IMPDH APO form complex. **c**, Angular distribution for particles of the *Msm*IMPDH APO form complex visualized on a globe-like plane. **d**, Fourier shell correlation (FSC) curves for the *Msm*IMPDH APO form complex. The plot of the FSC between two independently refined half-maps shows the overall resolution of the two maps as indicated by the gold standard FSC 0.143 cut-off criteria[2]. **e**, Surface representation of local resolution distribution of the *Msm*IMPDH APO form complex. The map is colored according to the local resolution calculated within the RELION 4.0[3] software package. Resolution is as indicated in the color bar.

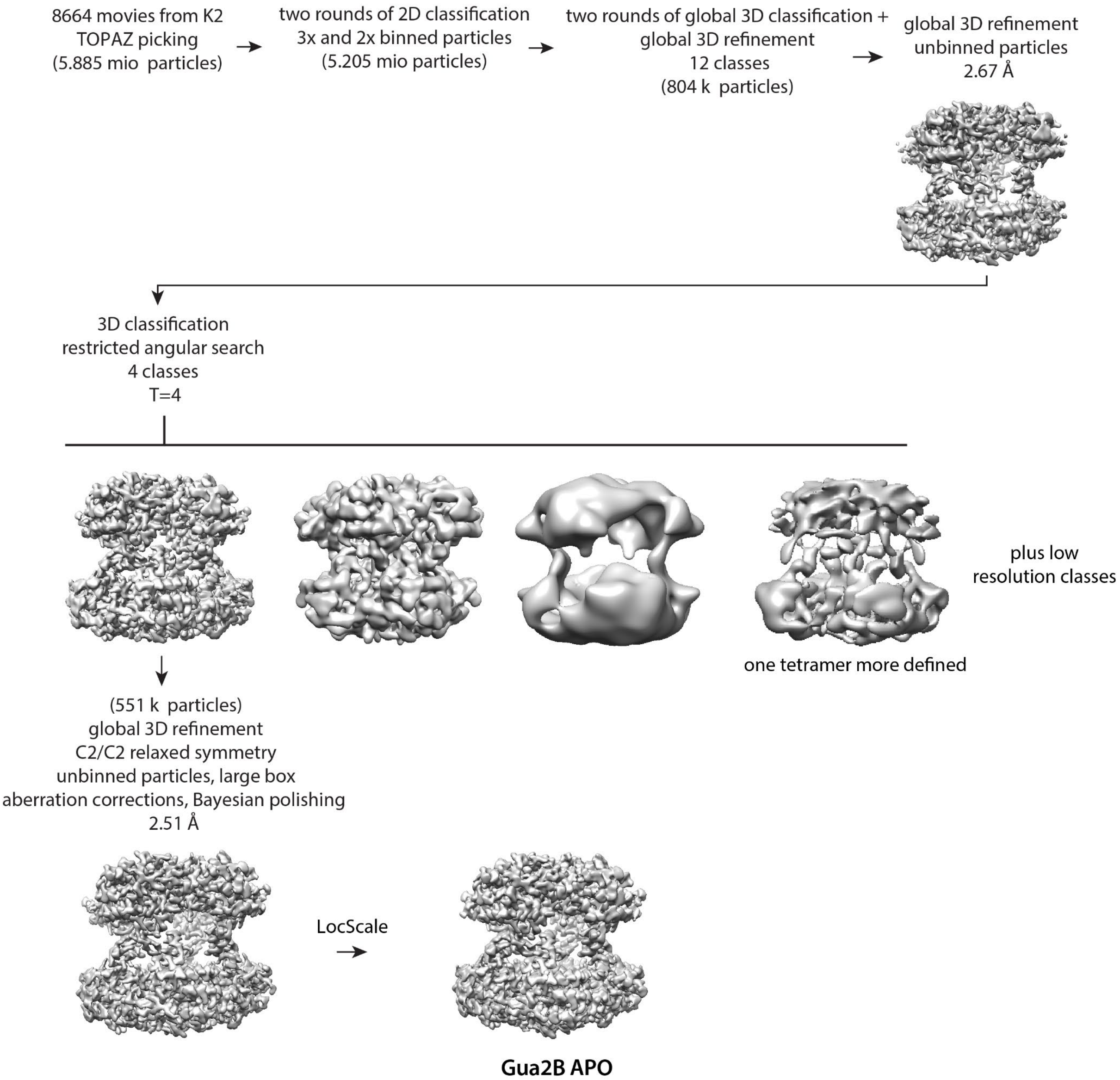

Figure EM-2: Cryo-EM data 3D classification and refinement scheme of the *Msm*IMPDH APO form complex

Final cryo-EM map was refined and post-processed with a respective mask in RELION 4.0[3] and filtered by LocScale[4].

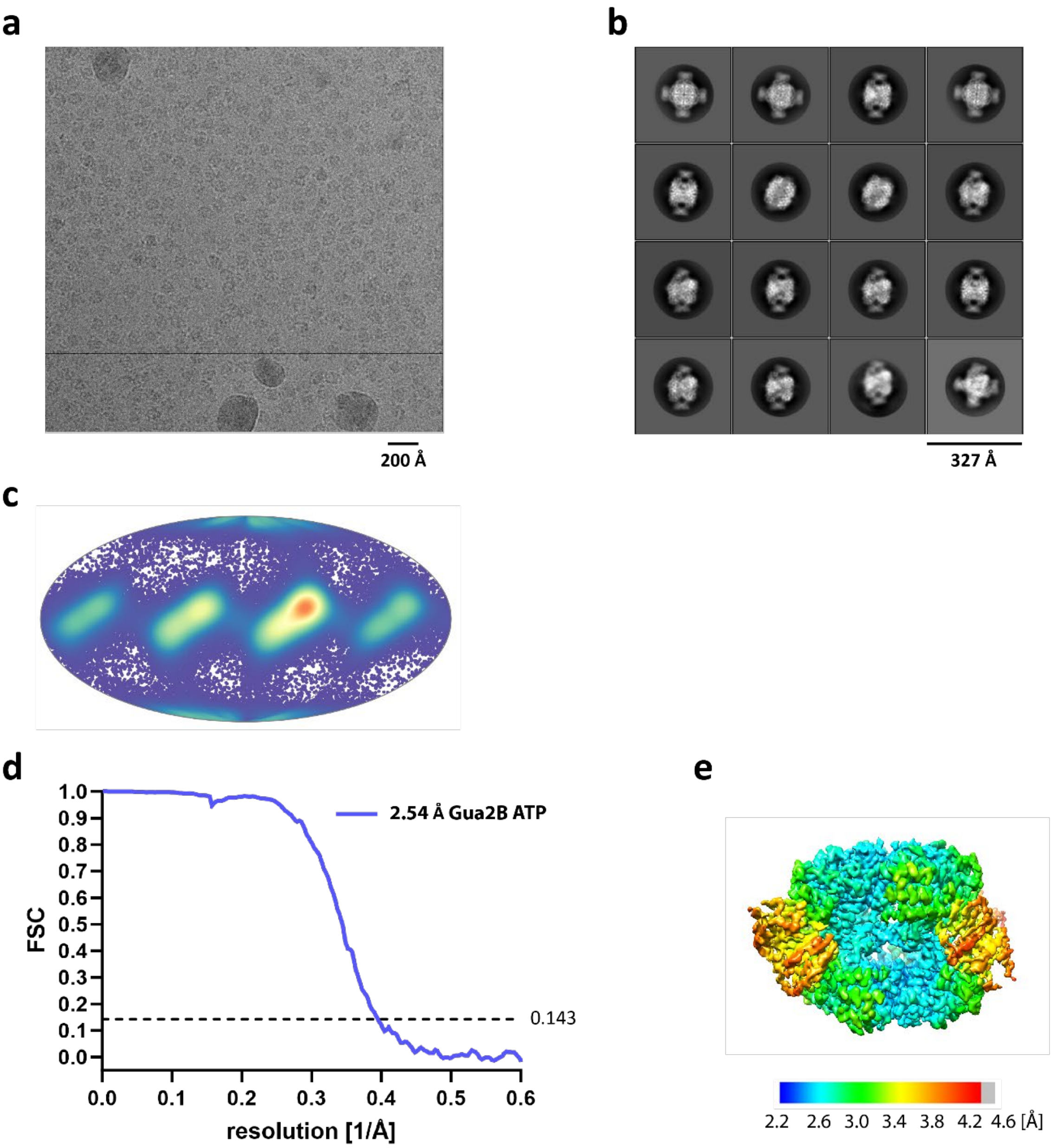

Figure EM-3: Cryo-EM of the *Msm*IMPDH ATP-bound complex

a, Micrograph of the *Msm*IMPDH ATP-bound complex in free standing ice after MotionCor2[1] correction at defocus of ~2.5 µm. **b**, 2D-class averages of the *Msm*IMPDH ATP-bound complex. **c**, Angular distribution for particles of the *Msm*IMPDH ATP-bound complex visualized on a globe-like plane. **d**, FSC curves for the *Msm*IMPDH ATP-bound complex. The plot of the FSC between two independently refined half-maps shows the overall resolution of the two maps as indicated by the gold standard FSC 0.143 cut-off criteria[2]. **e**, Surface representation of local resolution distribution of the *Msm*IMPDH ATP-bound complex. The map is colored according to the local resolution calculated within the RELION 4.0[3] software package. Resolution is as indicated in the color bar.

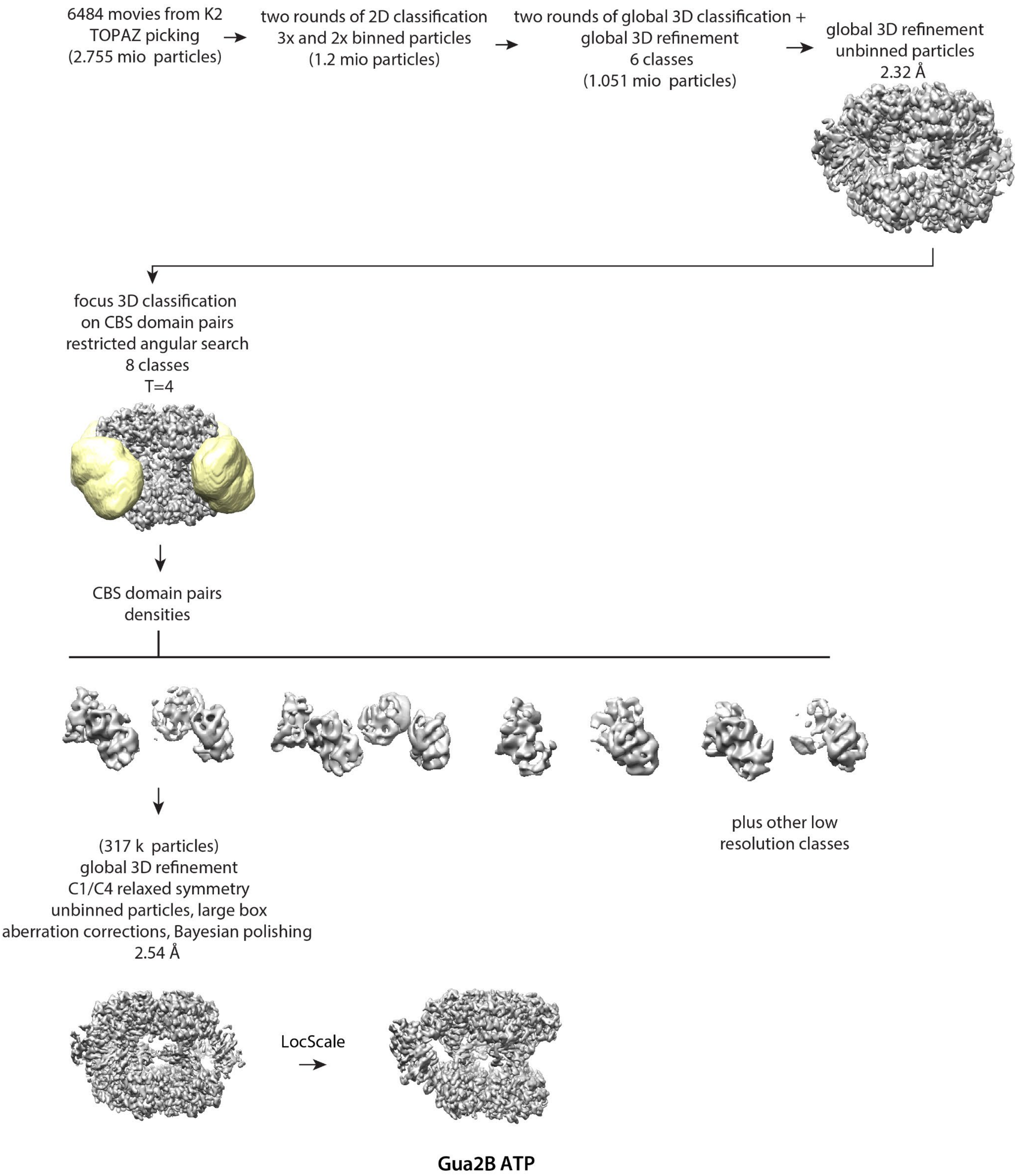

Figure EM-4: Cryo-EM data 3D classification and refinement scheme of the *Msm*IMPDH ATP-bound complex

Final cryo-EM map was refined and post-processed with a respective mask in RELION 4.0[3] and filtered by LocScale[4].

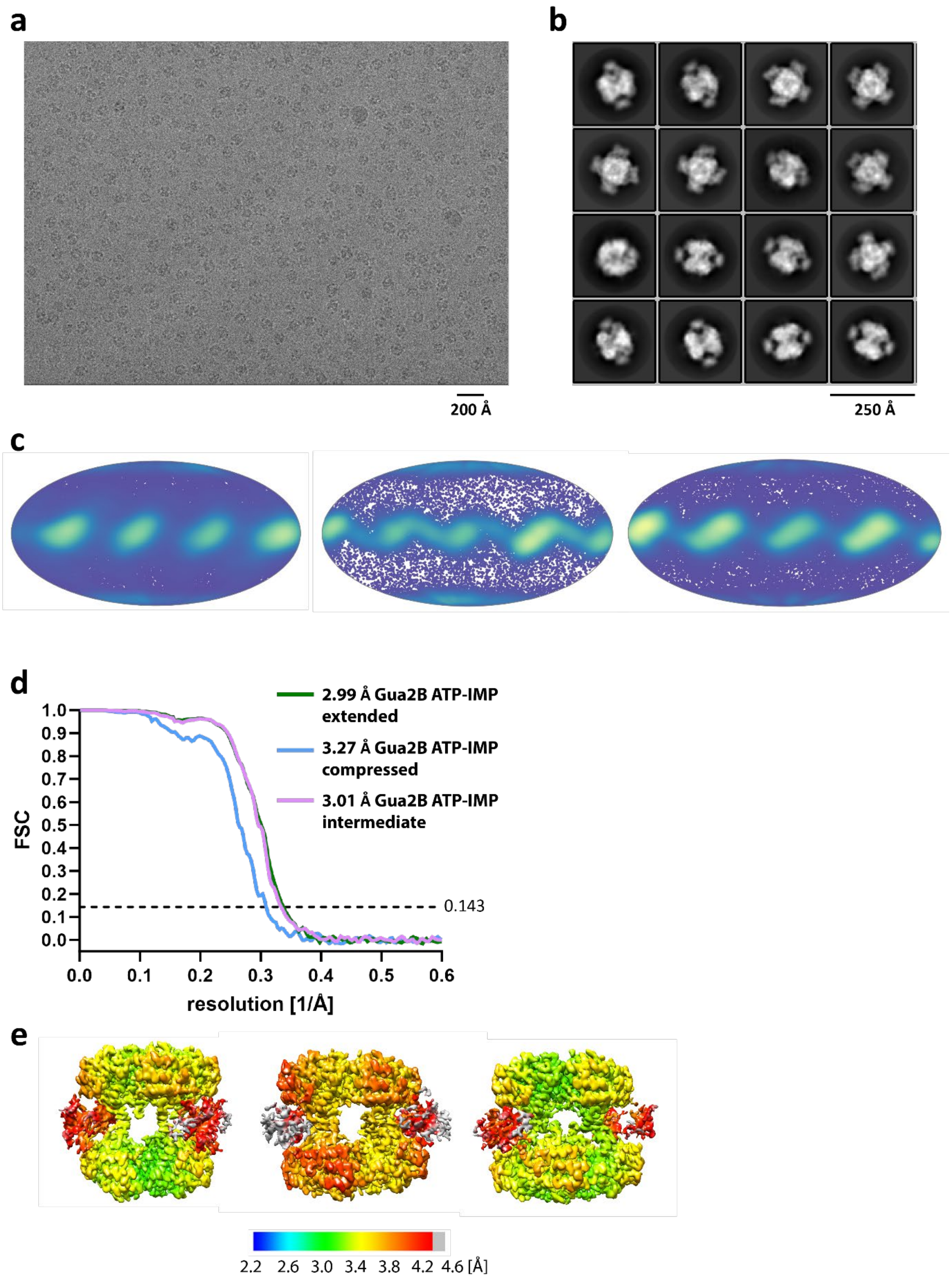

Figure EM-5: Cryo-EM of the *Msm*IMPDH ATP-IMP-bound complexes

a, Micrograph of the *Msm*IMPDH ATP-IMP-bound complexes in free standing ice after MotionCor2[1] correction at defocus of ~2.5 µm. **b**, 2D-class averages of the *Msm*IMPDH ATP-IMP-bound complexes. **c**, Angular distribution for particles of the *Msm*IMPDH ATP-IMP bound complexes in extended, compressed and intermediate form, respectively, visualized on a globe-like plane. **d**, FSC curves for the *Msm*IMPDH ATP-IMP-bound complexes. The plot

of the FSC between two independently refined half-maps shows the overall resolution of the two maps as indicated by the gold standard FSC 0.143 cut-off criteria[2]. **e**, Surface representation of local resolution distribution of the *Msm*IMPDH ATP-IMP-bound complexes in extended, compressed and intermediate form, respectively. The maps are colored according to the local resolution calculated within the RELION 4.0[3] software package. Resolution is as indicated in the color bar.

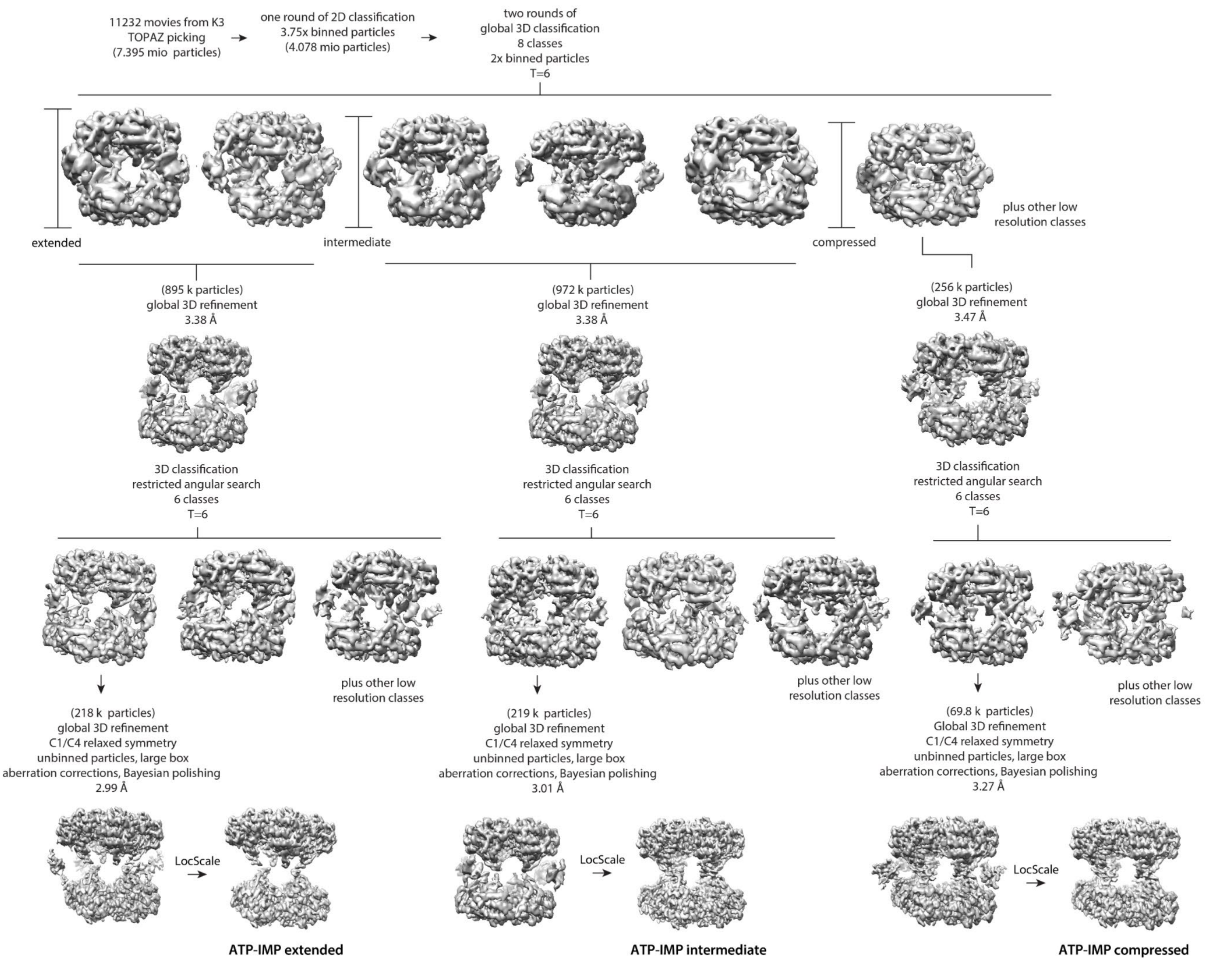

Figure EM-6: Cryo-EM data 3D classification and refinement scheme of the *Msm*IMPDH ATP-IMP bound complexes

Final cryo-EM maps were refined and post-processed with a respective mask in RELION 4.0[3] and filtered by LocScale[4].

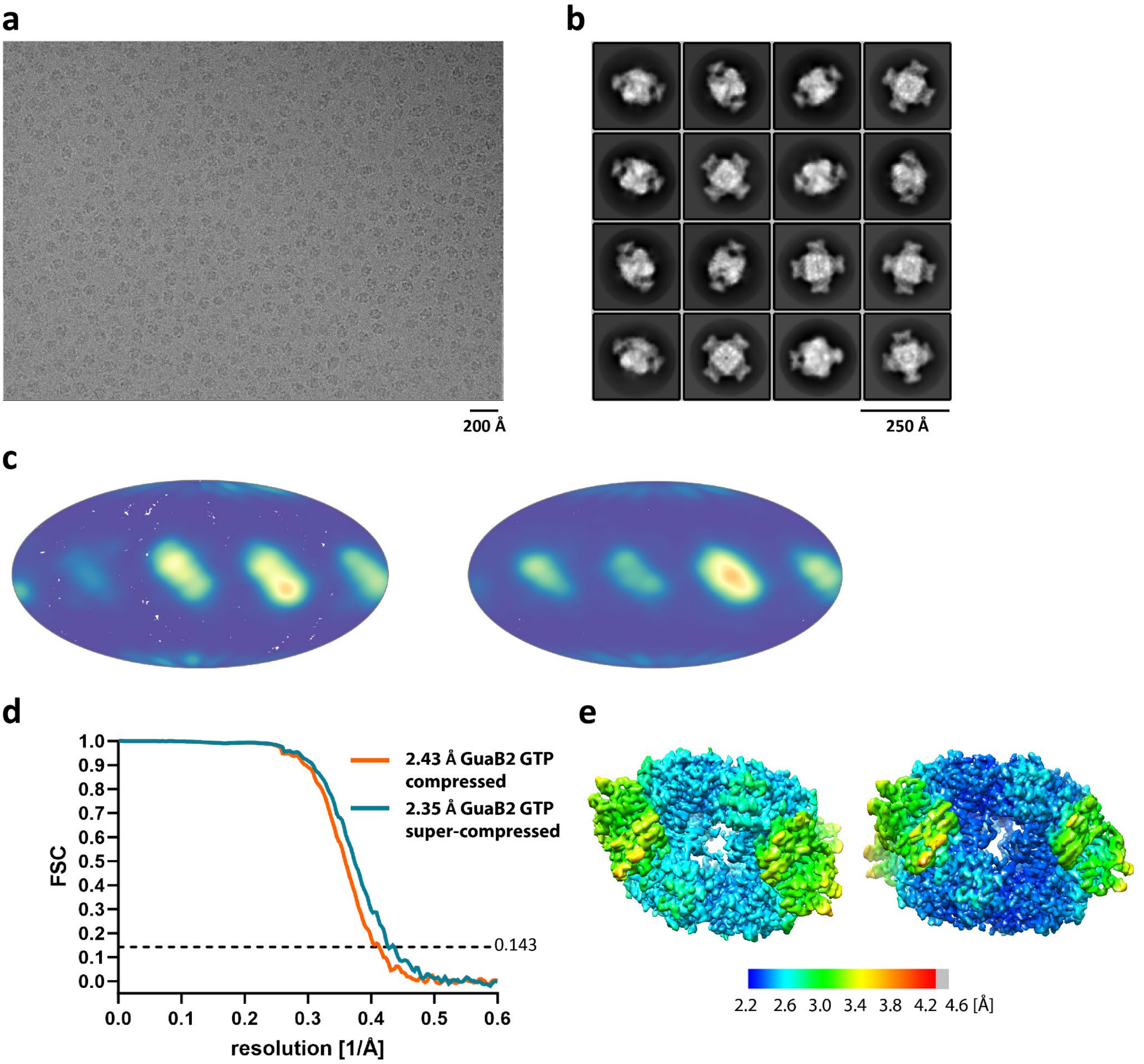

Figure EM-7: Cryo-EM of the *Msm*IMPDH GTP-ATP-bound complexes

a, Micrograph of the *Msm*IMPDH GTP-ATP-bound complexes in free standing ice after MotionCor2[1] correction at defocus of ~2.5 µm. **b**, 2D-class averages of the *Msm*IMPDH GTP-ATP-bound complexes. **c**, Angular distribution for particles of the *Msm*IMPDH GTP-ATP-bound complexes in compressed and less compressed form, respectively, visualized on a globe-like plane. **d**, FSC curves for the *Msm*IMPDH GTP-ATP-bound complexes. The plot of the FSC between two independently refined half-maps shows the overall resolution of the two maps as indicated by the gold standard FSC 0.143 cut-off criteria[2]. **e**, Surface representation of local resolution distribution of the *Msm*IMPDH GTP-ATP-bound complexes in compressed and super-compressed form, respectively. The maps are colored according to the local resolution calculated within the RELION 4.0[3] software package. Resolution is as indicated in the color bar.

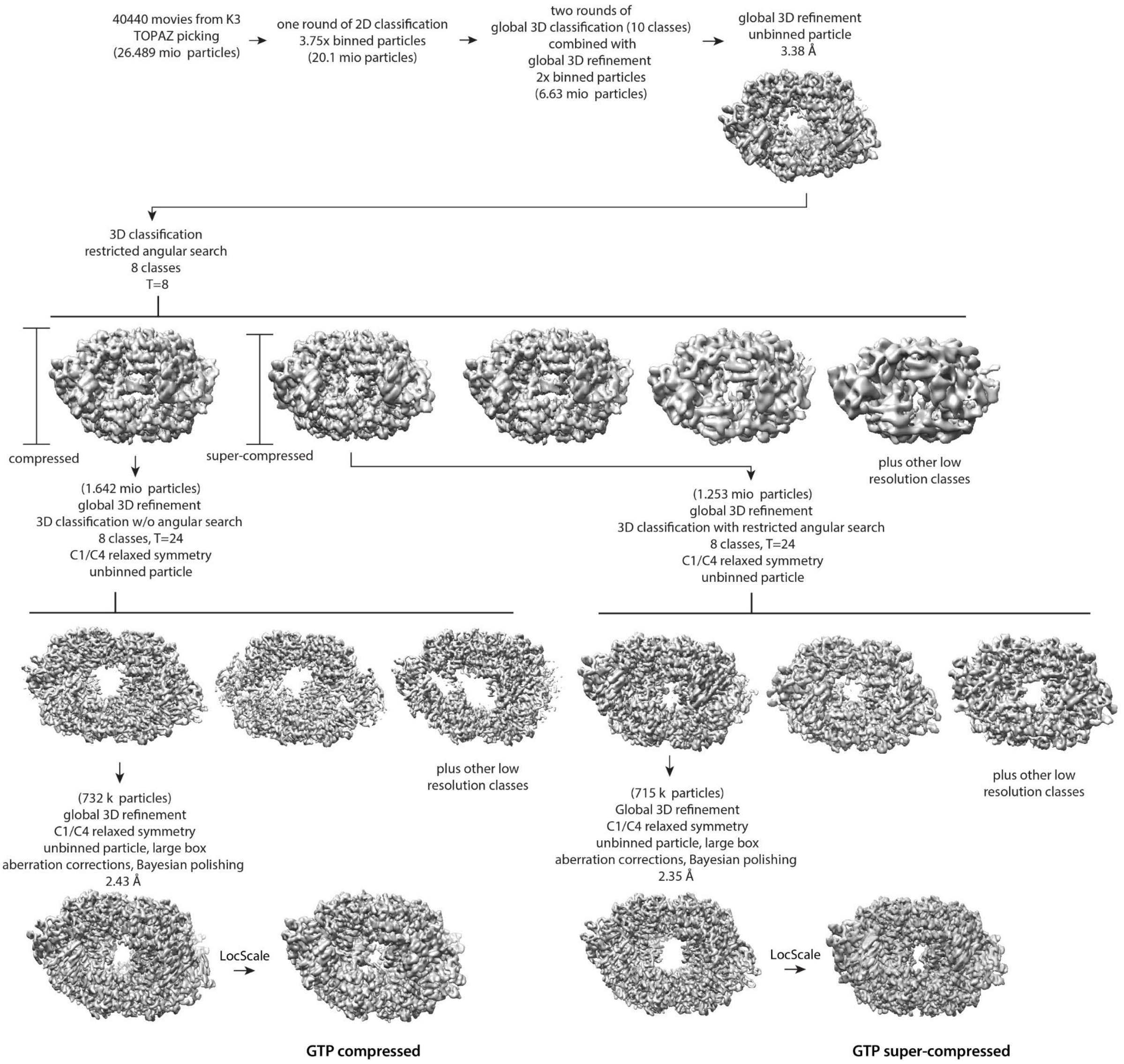

Figure EM-8: Cryo-EM data 3D classification and refinement scheme of the *Msm*IMPDH GTP-ATP bound complexes

Final cryo-EM maps were refined and post-processed with a respective mask in RELION 4.0[3] and filtered by LocScale[4].

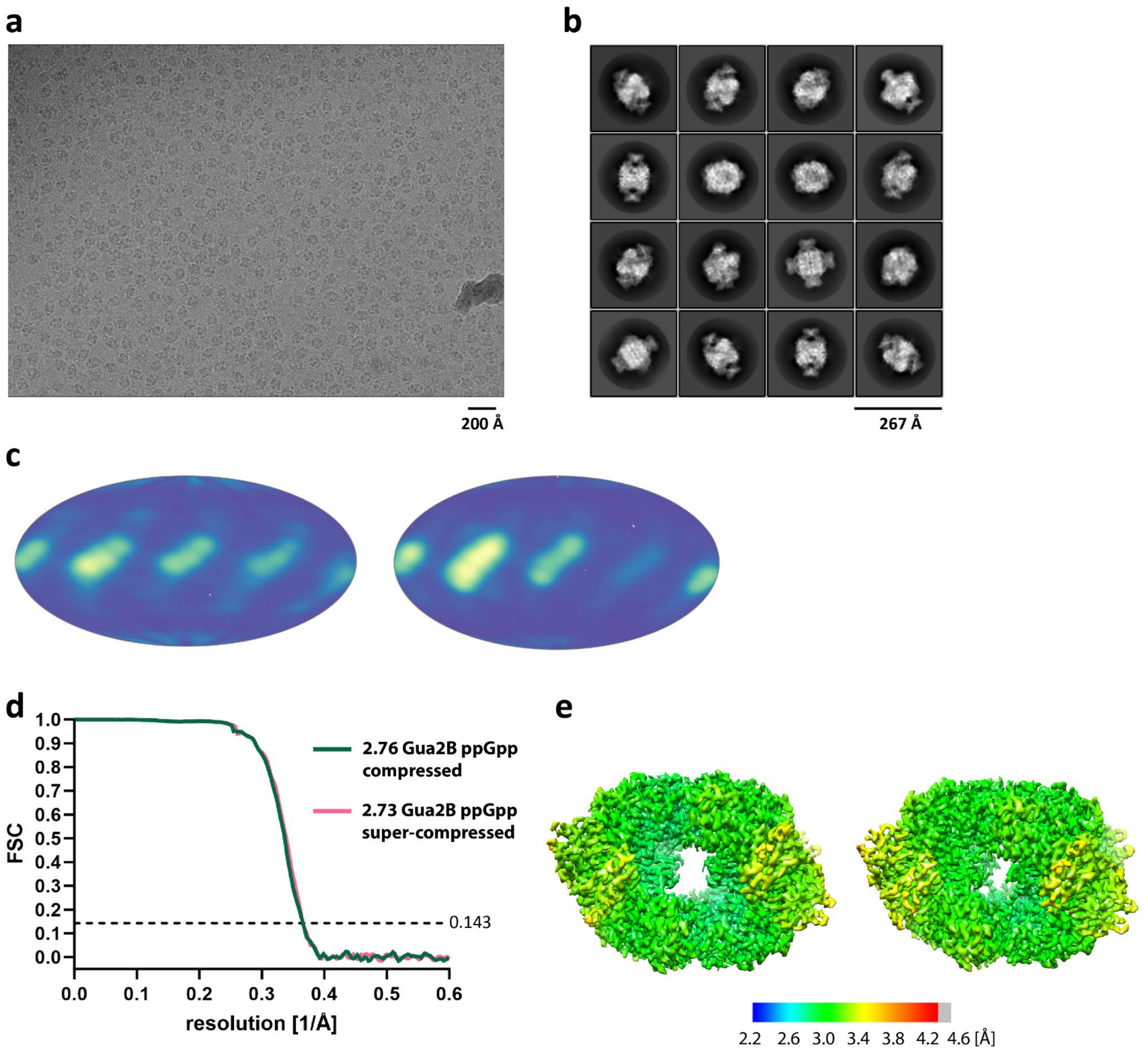

Figure EM-9: Cryo-EM of the *Msm*IMPDH ppGpp-ATP-bound complexes

a, Micrograph of the *Msm*IMPDH ppGpp-ATP-bound complexes in free standing ice after MotionCor2[1] correction at defocus of ~2.5 µm. **b**, 2D-class averages of the *Msm*IMPDH ppGpp-ATP-bound complexes. **c**, Angular distribution for particles of the *Msm*IMPDH ppGpp-ATP bound complexes in compressed and super-compressed form, respectively, visualized on a globe-like plane. **d**, FSC curves for the *Msm*IMPDH ppGpp-ATP-bound complex. The plot of the FSC between two independently refined half-maps shows the overall resolution of the two maps as indicated by the gold standard FSC 0.143 cut-off criteria[2]. **e**, Surface representation of local resolution distribution of the *Msm*IMPDH ppGpp-ATP-bound complexes in compressed and super-compressed form, respectively. The maps are colored according to the local resolution calculated within the RELION 4.0[3] software package. Resolution is as indicated in the color bar.

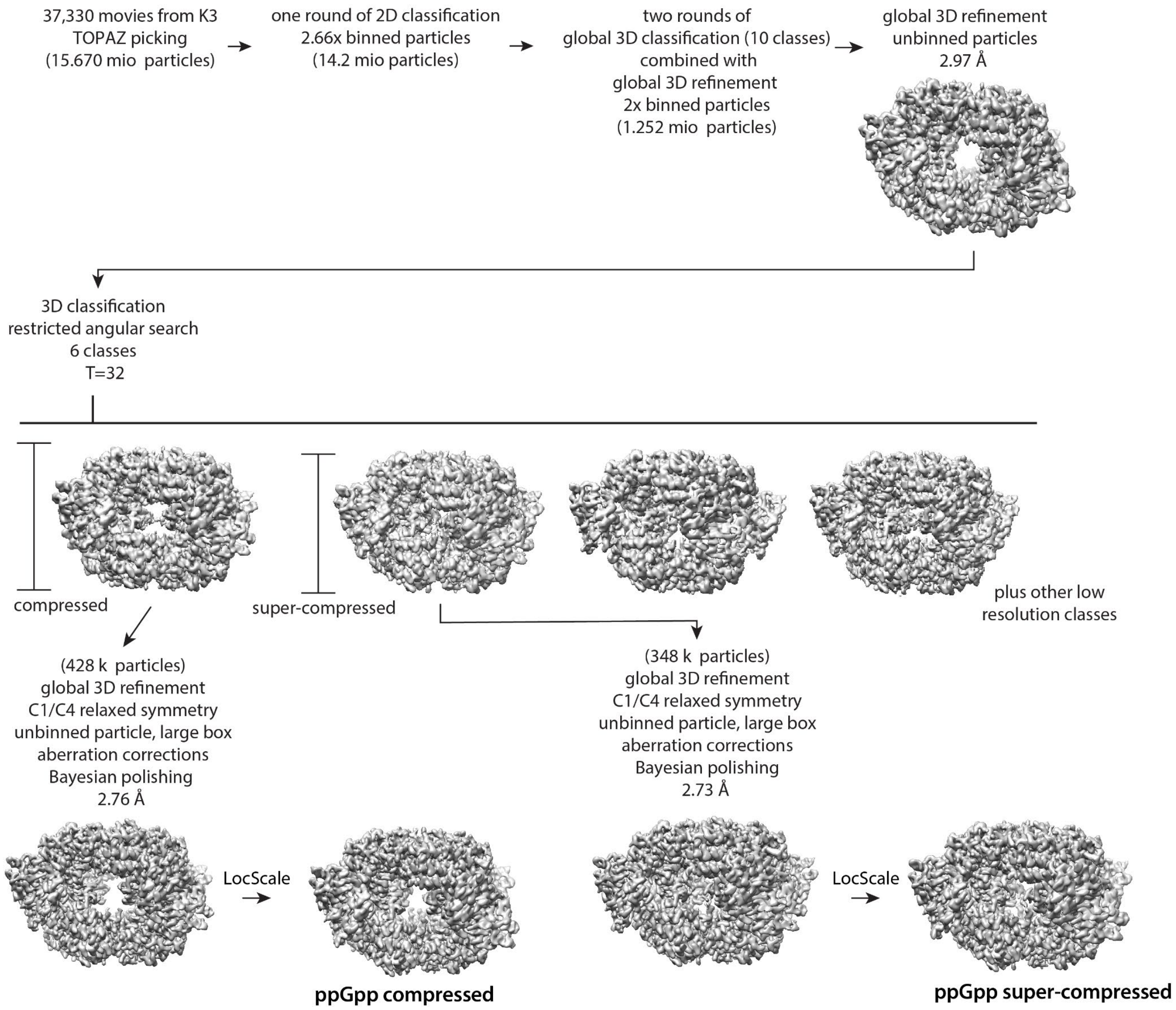

Figure EM-10: Cryo-EM data 3D classification and refinement scheme of the *Msm*IMPDH ppGpp-ATP bound complexes

Final cryo-EM maps were refined and post-processed with a respective mask in RELION 4.0[3] and filtered by LocScale[4].

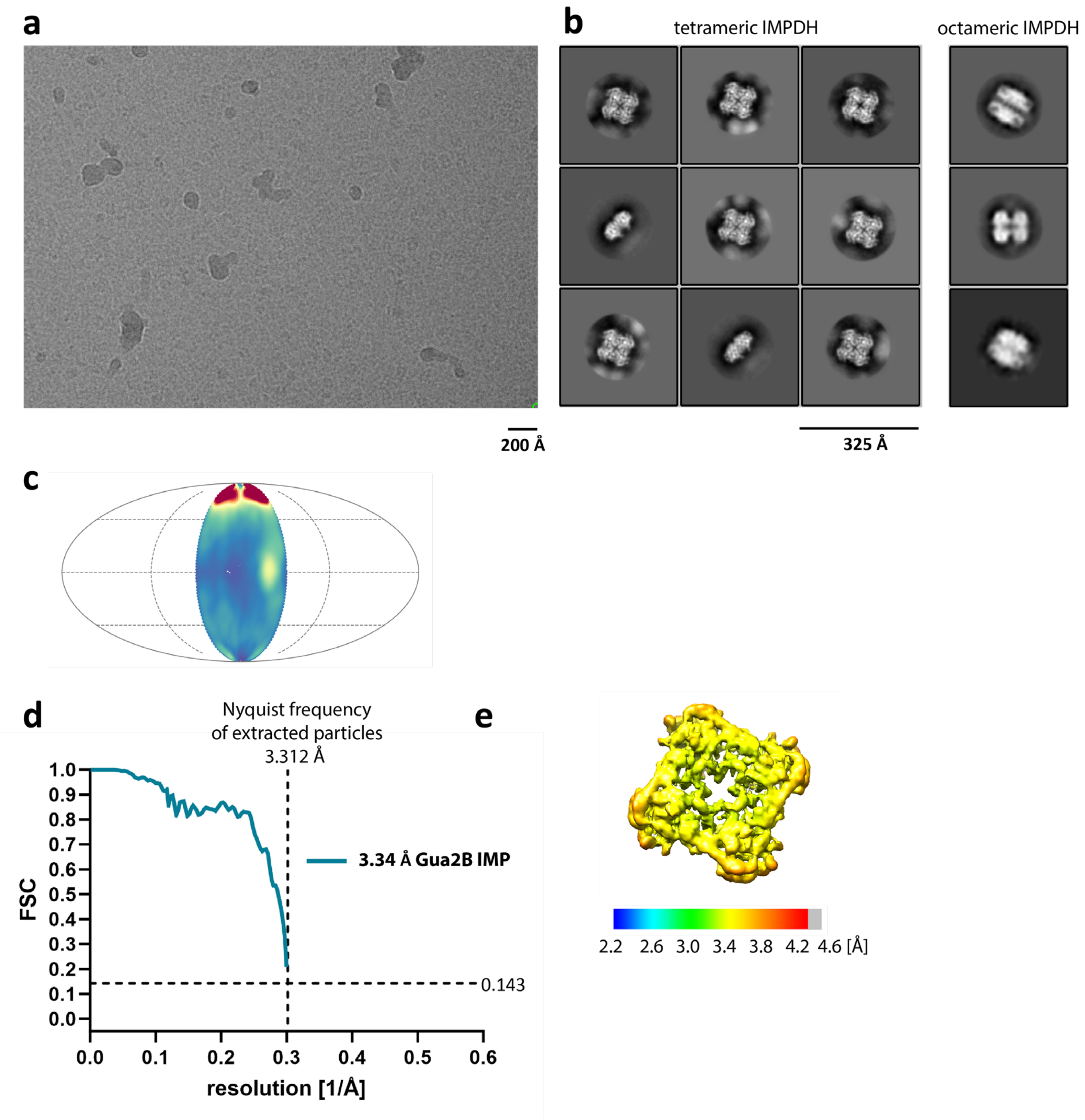

Figure EM-11: Cryo-EM of the *Msm*IMPDH IMP-bound complexes

a, Micrograph of the *Msm*IMPDH IMP-bound complex in free standing ice after MotionCor2[1] correction at defocus of ~2.5 µm. **b**, 2D-class averages of the *Msm*IMPDH IMP-bound complex. **c**, Angular distribution for particles of the *Msm*IMPDH IMP-bound complex in extended, compressed and intermediate form, respectively, visualized on a globe-like plane. **d**, FSC curves for the *Msm*IMPDH IMP-bound complex. The plot of the FSC between two independently refined half-maps shows the overall resolution of the two maps as indicated by the gold standard FSC 0.143 cut-off criteria[2]. **e**, Surface representation of local resolution distribution of the *Msm*IMPDH IMP-bound complex. The map is colored according to the local resolution calculated within the RELION 4.0[3] software package. Resolution is as indicated in the color bar.

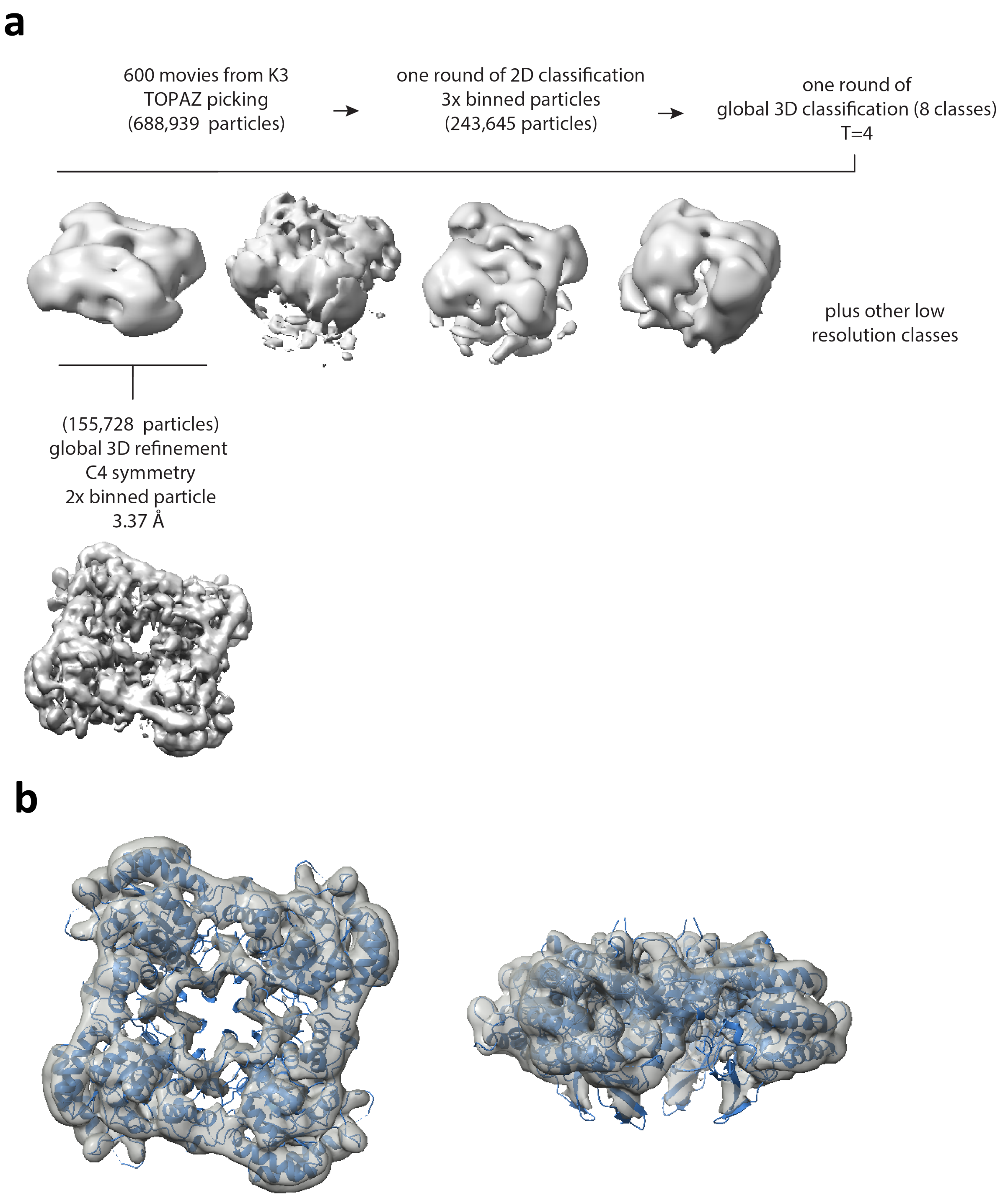

Figure EM-12: Cryo-EM data 3D classification scheme and model fitting of the *Msm*IMPDH IMP-bound complex

a, Summary of the cryo-EM 3D classification and refinement scheme of the *Msm*IMPDH IMP-bound complex. Final cryo-EM map was refined and post-processed with a respective mask in RELION 4.0[3] and filtered by LocScale[4].

b, Atomic model of the *Msm*IMPDH IMP-bound tetramer, derived from the ATP-IMP-bound complexes structures was rigid body fitted into cryoEM map by MolRep[5] within CCP-EM package[6].

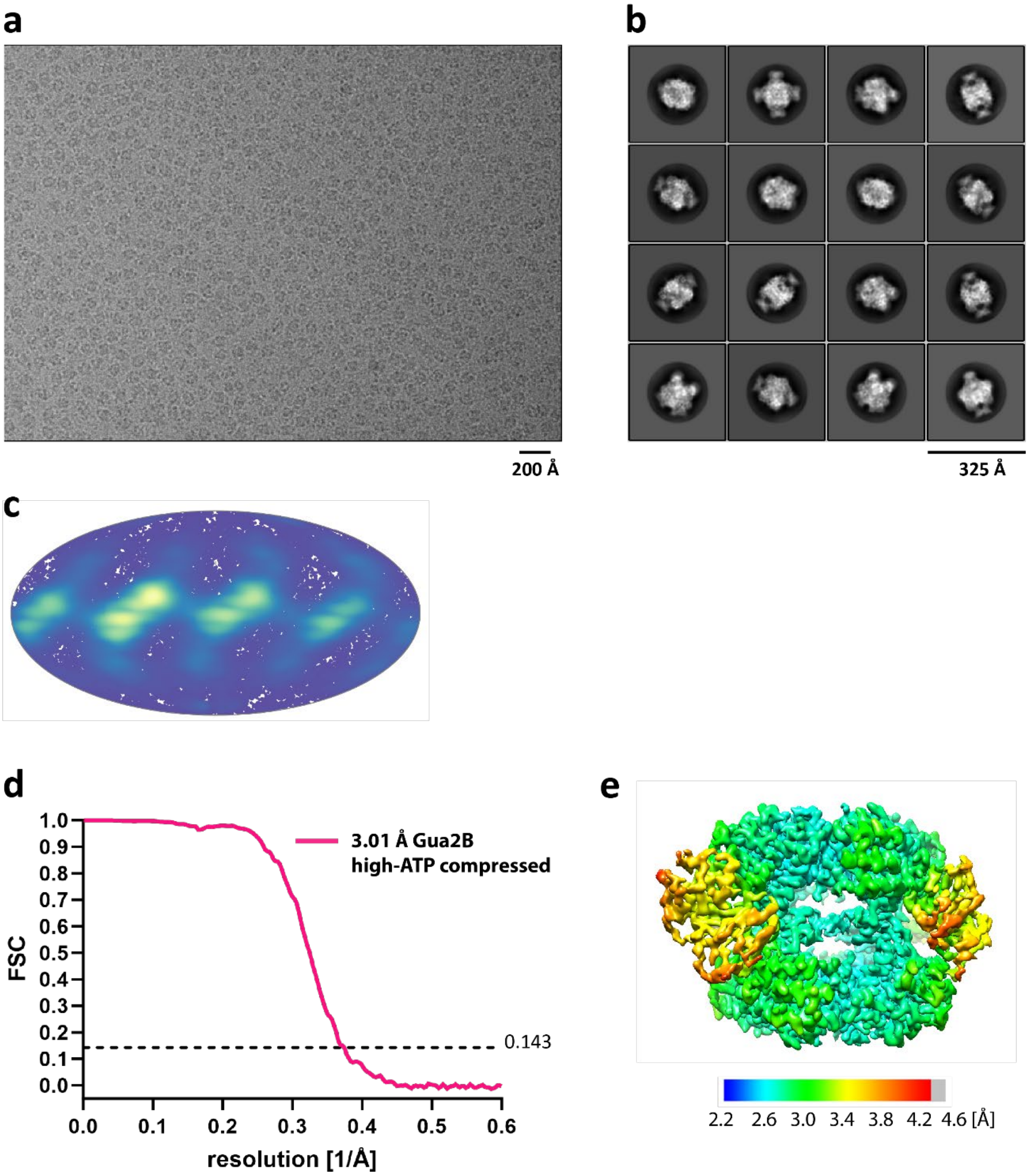

Figure EM-13: Cryo-EM of the *Msm*IMPDH high concentration ATP bound complexes

a, Micrograph of high concentration ATP bound complex in free standing ice after MotionCor2[1] correction at defocus of ~2.5 µm. **b**, 2D-class averages of the high concentration ATP bound complex. **c**, Angular distribution for particles of the high concentration ATP bound complex on a globe-like plane. **d**, FSC curves for the high concentration ATP bound complex. The plot of the FSC between two independently refined half-maps shows the overall resolution of the two maps as indicated by the gold standard FSC 0.143 cut-off criteria[2]. **e**, Surface representation of local resolution distribution of the high concentration ATP bound complex. The map is colored according to the local resolution calculated within the RELION 4.0[3] software package. Resolution is as indicated in the color bar.

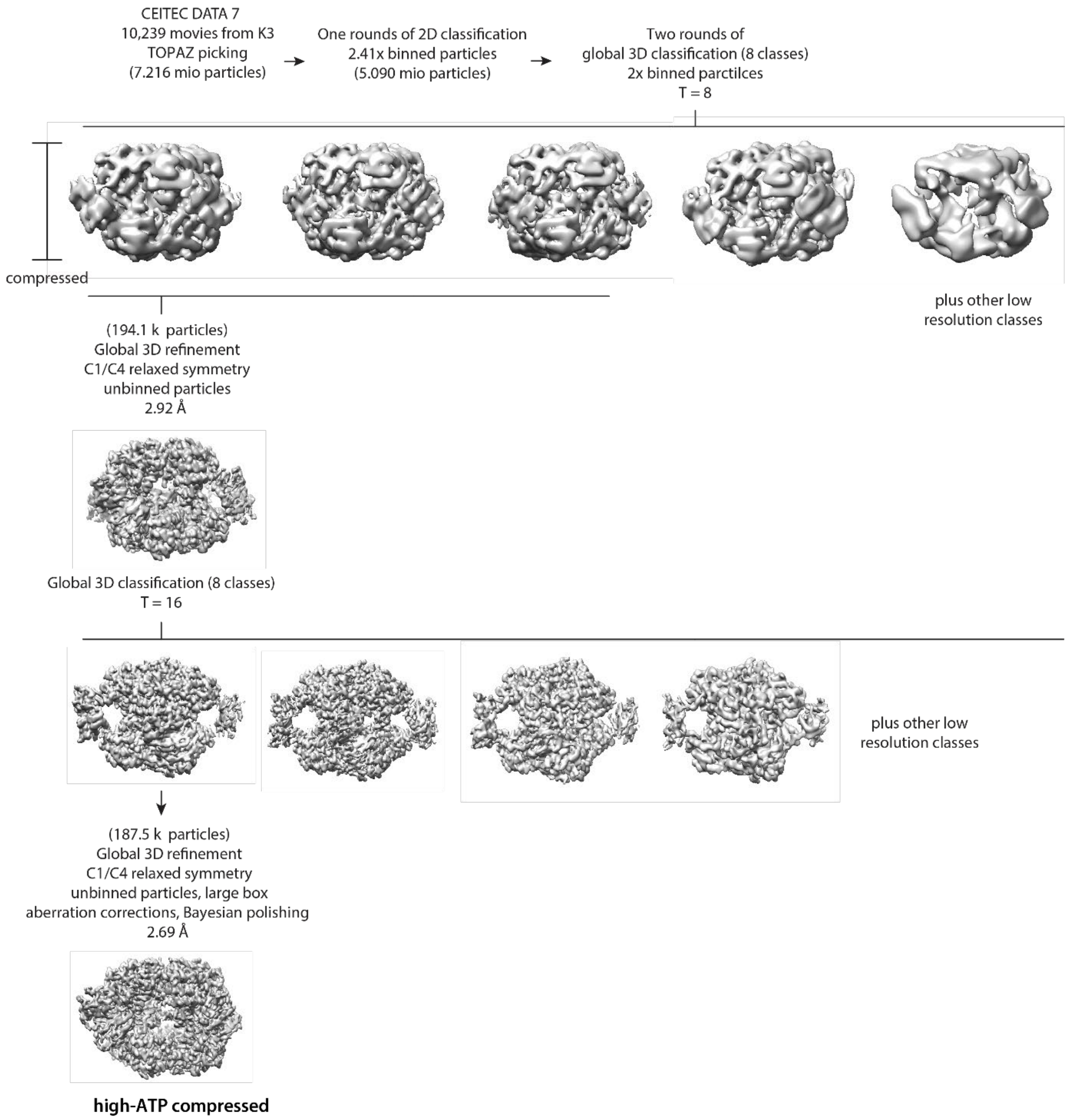

Figure EM-14: Cryo-EM data 3D classification and refinement scheme of the *Msm*IMPDH high concentration ATP bound complexes

Final cryo-EM map was refined and post-processed with a respective mask in RELION 4.0[3] and filtered by LocScale[4].

Supplementary SAXS data

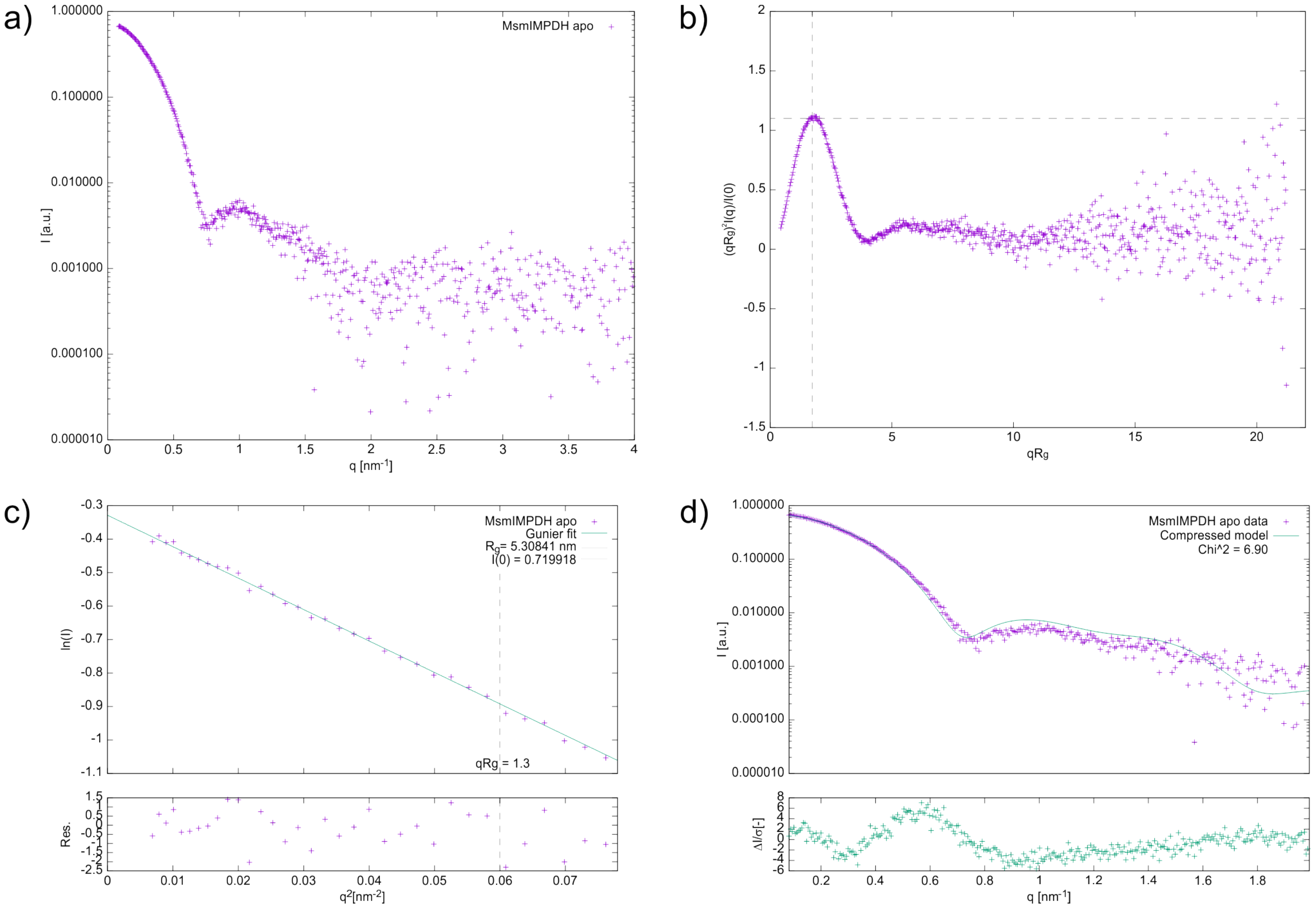

Figure SAXS-1: SAXS results of the *Msm*IMPDH in apo form

a, Experimental SAXS profile of *Msm*IMPDH in apo form. **b**, Dimensionless Kratky plot. The dashed lines represent the peak position $qR_g=1.73$ and peak height $3/e=1.1$, which are values typical for globular proteins. **c**, Guinier fit for $qR_g < 1.3$ values. The lower panel shows the error-weighted residual difference plot. **d**, Model fit for the theoretical SAXS profile of the compressed octamer of *Msm*IMPDH calculated by CRYSOL (green solid line). The lower panel shows the error-weighted residual difference plot $\Delta/\sigma = [I_{exp}(q) - cI_{mod}(q)]/\sigma(q)$ versus q.

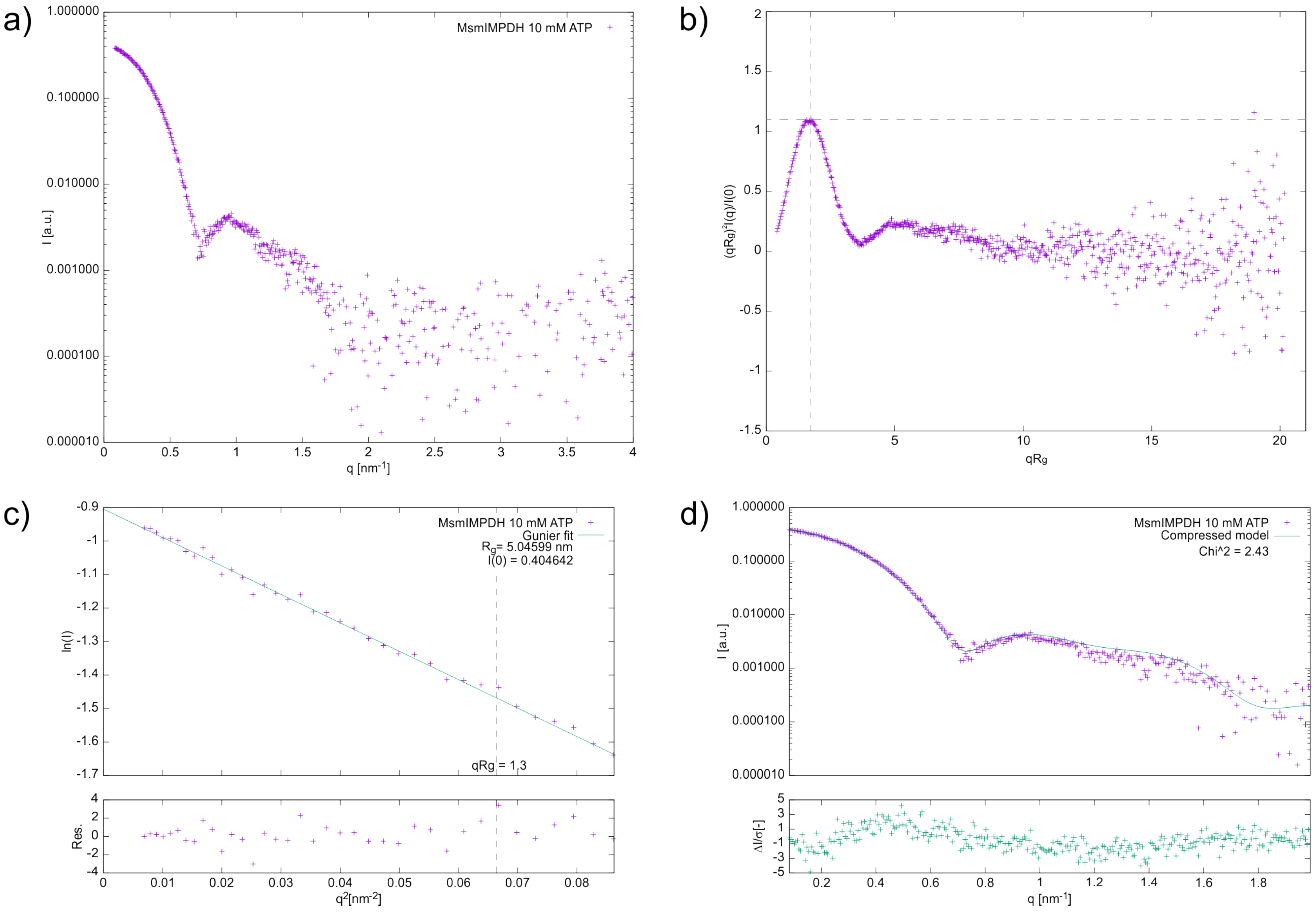
a)
MsmIMPDH 10 mM ATP
I [a.u.]
q [nm^{-1}]
b)
$(qR_g)^2 I(q)/I(0)$
qR_g
c)
MsmIMPDH 10 mM ATP
Gunier fit
R_g= 5.04599 nm
I(0) = 0.404642
qRg = 1.3
ln(I)
Res.
q^2[nm^{-2}]
d)
MsmIMPDH 10 mM ATP
Compressed model
Chi^2 = 2.43
I [a.u.]
ΔI/σ[-]
q [nm^{-1}]

Figure SAXS-2: SAXS results of the *Msm*IMPDH in ATP-bound form

a, Experimental SAXS profile of *Msm*IMPDH with 10 mM ATP. **b**, Dimensionless Kratky plot. The dashed lines represent the peak position qR_g=1.73 and peak height 3/*e*=1.1, which are values typical for globular proteins. The lower panel shows the error-weighted residual difference plot. **d**, Model fit for the theoretical SAXS profile of the compressed octamer of *Msm*IMPDH calculated by CRYSOL (green solid line). The lower panel shows the error-weighted residual difference plot $\Delta/\sigma = [I_{exp}(q) - cI_{mod}(q)]/\sigma(q)$ versus q.

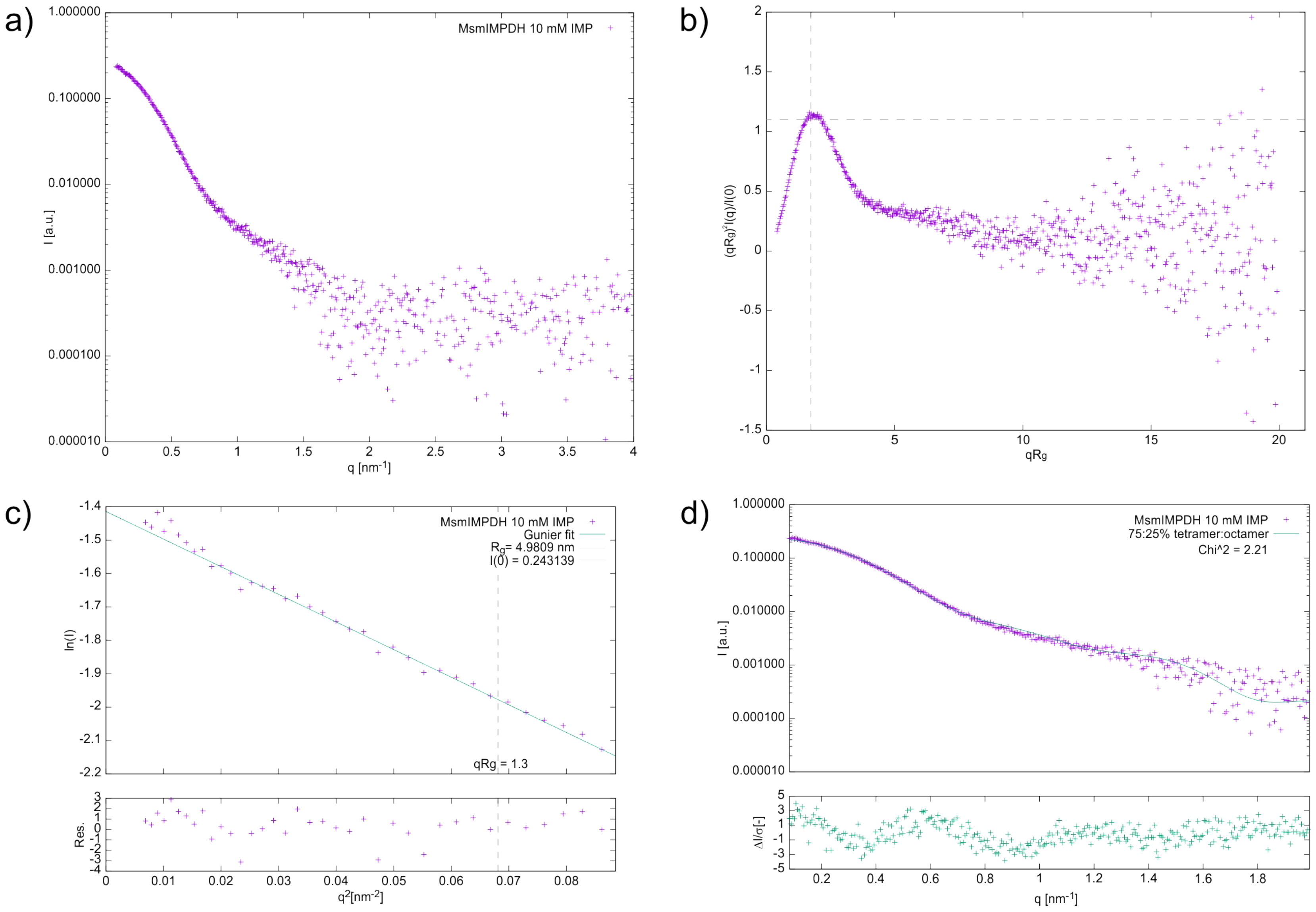
a)
MsmIMPDH 10 mM IMP
I [a.u.]
q [nm-1]
b)
(qRg)²I(q)/I(0)
qRg
c)
MsmIMPDH 10 mM IMP
Gunier fit
Rg= 4.9809 nm
I(0) = 0.243139
qRg = 1.3
ln(I)
Res.
q²[nm-2]
d)
MsmIMPDH 10 mM IMP
75:25% tetramer:octamer
Chi^2 = 2.21
I [a.u.]
ΔI/σ[-]
q [nm-1]

Figure SAXS-3: SAXS results of the *Msm*IMPDH in IMP-bound form

a, Experimental SAXS profile of *Msm*IMPDH with 10 mM IMP. **b**, Dimensionless Kratky plot. The dashed lines represent the peak position qR_g=1.73 and peak height 3/*e*=1.1, which are values typical for globular proteins. The lower panel shows the error-weighted residual difference plot. **d**, Model fit for the theoretical SAXS profile of 75:25% tetramer:octamer combination of *Msm*IMPDH calculated by OLIGOMER (green solid line). The lower panel shows the error-weighted residual difference plot $\Delta/\sigma = [I_{exp}(q) - cI_{mod}(q)]/\sigma(q)$ versus q.

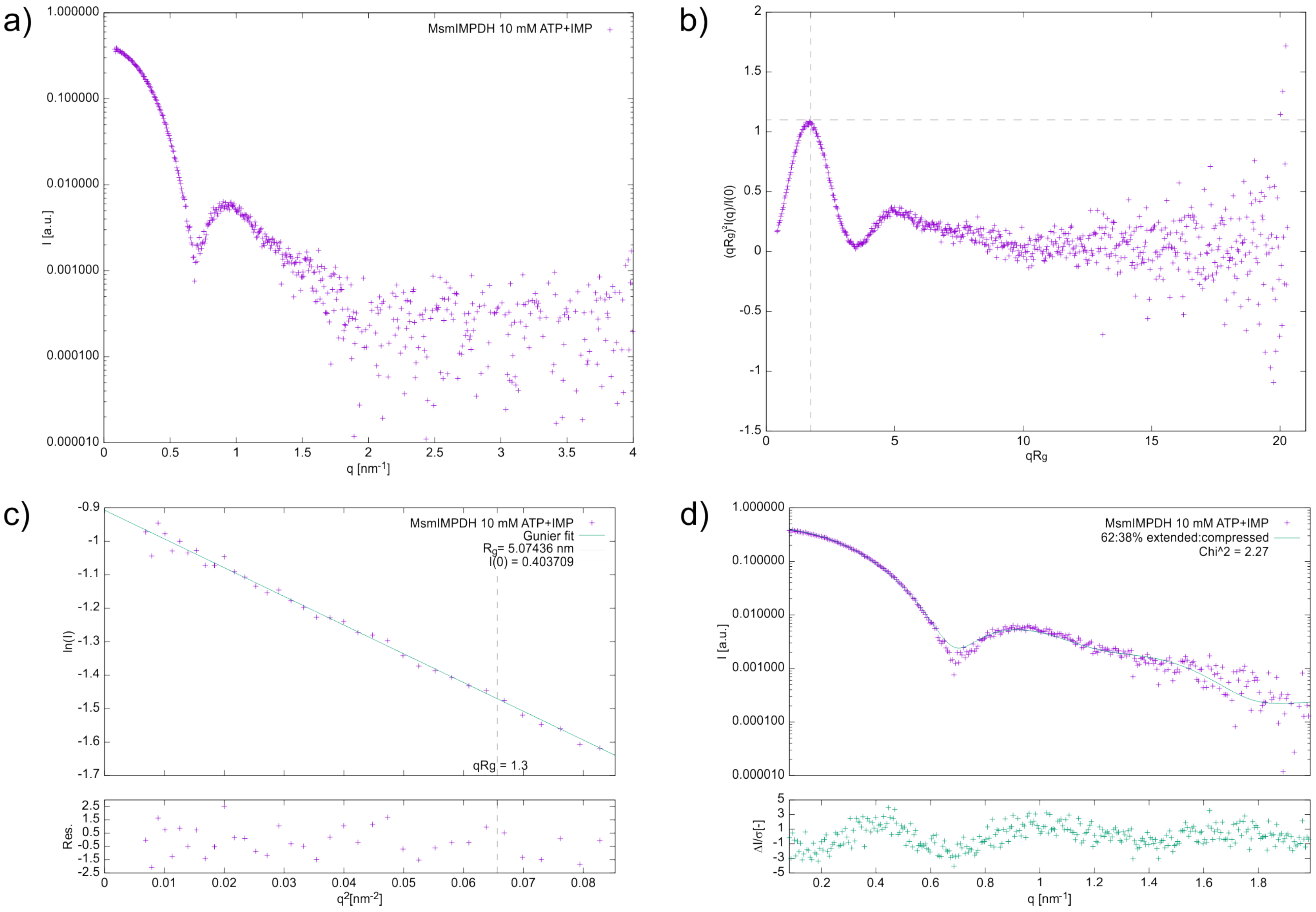

a)
MsmIMPDH 10 mM ATP+IMP
I [a.u.]
q [nm-1]
b)
(qRg)²I(q)/I(0)
qRg
c)
MsmIMPDH 10 mM ATP+IMP
Gunier fit
Rg= 5.07436 nm
I(0) = 0.403709
qRg = 1.3
ln(I)
Res.
q²[nm-2]
d)
MsmIMPDH 10 mM ATP+IMP
62:38% extended:compressed
Chi^2 = 2.27
I [a.u.]
ΔI/σ[-]
q [nm-1]

Figure SAXS-4: SAXS results of the *Msm*IMPDH in ATP+IMP-bound form

a, Experimental SAXS profile of *Msm*IMPDH with 10 mM ATP and IMP. **b**, Dimensionless Kratky plot. The dashed lines represent the peak position qR_g=1.73 and peak height 3/*e*=1.1, which are values typical for globular proteins. The lower panel shows the error-weighted residual difference plot. **d**, Model fit for the theoretical SAXS profile of 62:38% extended:compressed octamer of *Msm*IMPDH combination calculated by OLIGOMER (green solid line). The lower panel shows the error-weighted residual difference plot $\Delta/\sigma = [I_{exp}(q) - cI_{mod}(q)]/\sigma(q)$ versus q.

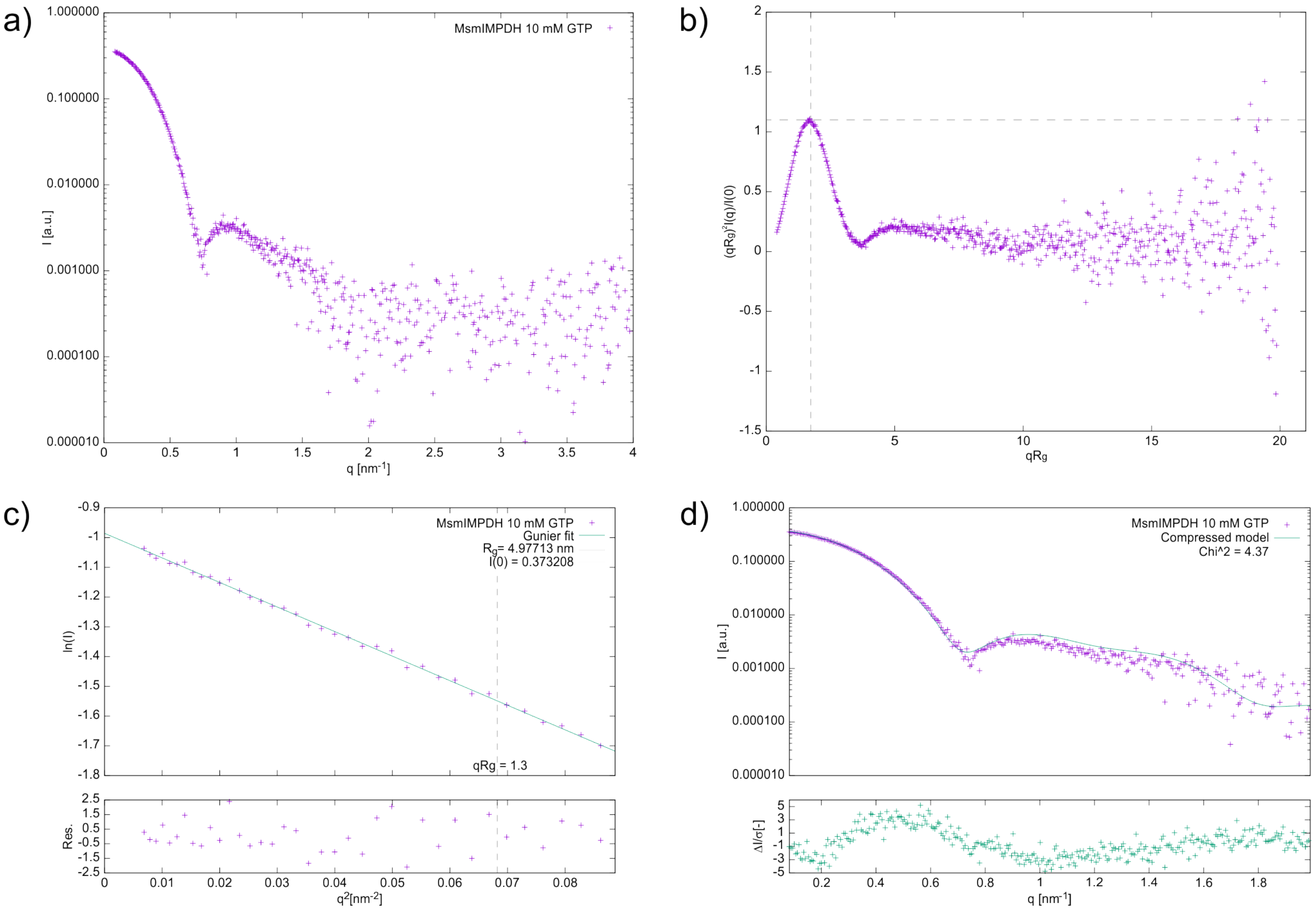

a)
MsmIMPDH 10 mM GTP
I [a.u.]
q [nm-1]
b)
(qRg)²I(q)/I(0)
qRg
c)
MsmIMPDH 10 mM GTP
Gunier fit
Rg= 4.97713 nm
I(0) = 0.373208
qRg = 1.3
ln(I)
Res.
q²[nm-2]
d)
MsmIMPDH 10 mM GTP
Compressed model
Chi^2 = 4.37
I [a.u.]
ΔI/σ[-]
q [nm-1]

Figure SAXS-5: SAXS results of the *Msm*IMPDH in GTP-bound form

a, Experimental SAXS profile of *Msm*IMPDH with 10 mM GTP. **b**, Dimensionless Kratky plot. The dashed lines represent the peak position qR_g=1.73 and peak height 3/*e*=1.1, which are values typical for globular proteins. **c**, Guinier fit for $qR_g < 1.3$ values. The lower panel shows the error-weighted residual difference plot. **d**, Model fit for the theoretical SAXS profile of the compressed octamer of *Msm*IMPDH calculated by CRYSOL (green solid line). The lower panel shows the error-weighted residual difference plot $\Delta/\sigma = [I_{exp}(q) - cI_{mod}(q)]/\sigma(q)$ versus q.

Supplementary Methods

Nucleotides

Sodium salts of ATP, ADP, IMP, and NAD^+ were purchased from Santa Cruz. A GTP solution of 100 mM was purchased from Roth. Nucleotide solutions of 50–100 mM were prepared in water, and the pH was immediately adjusted to a pH of 7.0 using 1 M NaOH. The exact concentration of the stocks was determined spectrophotometrically at the absorption maxima using the following absorption coefficients: $\varepsilon^{260\ nm}$ (ATP) 15.4 $mM^{-1}cm^{-1}$; $\varepsilon^{253\ nm}$ (GTP, GDP) 13.7 $mM^{-1}cm^{-1}$; $\varepsilon^{260\ nm}$ (NAD^+) 17.4 $mM^{-1}cm^{-1}$; $\varepsilon^{249\ nm}$ (IMP) 12.2 $mM^{-1}cm^{-1}$. The aliquoted stocks were stored at −70°C. The following molecules were obtained from Jena Biosciences in solutions of 10 or 20 mM: 3',5'-cyclic diguanylic acid (c-diGMP); 3',5'-cyclic diadenylic acid (c-diAMP); cyclic guanosine-2'5'-monophosphate-adenosine-3'5'-monophosphate (c-GAMP); P1-(5'-adenosyl) P4-(5'-guanosyl) tetraphosphate (Ap4G); and P1-(5'-adenosyl) P4-(5'-adenosyl) tetraphosphate (Ap4A). ppGpp and pppGpp were synthesized enzymatically, as previously described[7]. pGpp and ppApp were synthesized chemically, as previously described[8,9].

Detailed conditions and parameters of LC–MS analysis

Peptides were identified by tandem mass spectrometry of non-deuterated protein. Samples were injected into a refrigerated nanoAcquity UPLC M-Class System (Waters), with chromatographic elements held at 0 °C. Samples were passed through a protease column with co-immobilized pepsin and nepenthesin-2 proteases (Affipro, AP-PC-006, 2.1 mm x 20 mm) at 15 °C with a flow rate of 100 µl min^{-1} (0.1% v/v FA). The generated peptides were trapped and desalted for 3 min on an Acquity UPLC BEH C18 VanGuard pre-column (Waters, 2.1 mm x 5 mm, pore size 1.7µm). Subsequently, peptides were separated over an Acquity UPLC BEH C18 column (1 mm x 100 mm, pore size 1.7 µm) for 12 min (10–35% CH_3CN v/v and 0.1% v/v FA, flow rate 40 µl min^{-1}). MS spectra were acquired using the Synapt G2-Si mass spectrometer (Waters) configured with electrospray ionization (ESI) and quadrupole/time-of-flight (Q/TOF) within a mass range of 50 to 2000 m/z; scans were performed every 0.4 s. Leucine enkephalin served as the continuous (lock-spray) calibration standard.

Detailed parameters for MS data processing using Waters software

ProteinLynx Global Server (PLGS) v.3.0.2

The PLGS processing parameters for MS/MS data were as follows: chromatographic peak width – automatic; MS TOF resolution – automatic; lock mass for charge +1 – 556.2771 Da/e; lock-mass window – 0.25 Da; low-energy threshold – 135.0 counts; elevated energy threshold – 30.0 count; and intensity threshold – 750.0 counts. PLGS workflow parameters were as follows: fasta file search containing forward and reverse sequences of the examined protein, namely pepsin (UniProt code P00791) and nepenthesin-2; peptide tolerance and fragment tolerance – automatic; minimum fragment ion matches per peptide – 3; minimum fragment ion matches per protein – 7; minimum peptide matches per protein – 1; primary digest reagent – non-specific; number of missed cleavages – 3; oxidation of methionines as a variable modifier reagent; false discovery rate – 5; monoisotopic mass of peptides with charge +1 used for the ESI-QUAD-TOF instrument type.

DynamX v.3.0

DynamX v.3.0 (Waters) was used to filter peptides, selecting those presenting 0.3 fragments per amino acid and a mass error for the precursor ion below 10 ppm. In addition, only peptides identified in at least 3 out of 5 acquired MS/MS files, with a maximum length of up to 25 amino

acids, a minimum signal intensity of 3000, and a retention time of up to 5 % RSD, were used for further analysis.

For processing of MS files, the following parameters were used: both chromatographic peak width and MS TOF resolution were set as automatic, with 556.2771 Da used as a lock mass for charge +1, a lock-mass window of 0.25 Da, a low-energy threshold of 130, and an elution time range of 2.5–9 min to conduct the data search. DynamX advanced processing parameters were not applied.

Statistics and visualization of results in HDX–MS experiments

DynamX 3.0 was used to calculate the deuterium contents of peptides generated from labelled proteins. Statistically significant differences in deuterium incorporation between each two considered protein states at the peptide level were determined by applying the threshold for the 98% confidence interval. This was calculated using the root-mean-square of the standard deviations of peptide deuterium contents for time points performed in triplicate, followed by application of the Student's *t*-test. Finally, the deuterium uptakes of common peptides were shown in exchange plots to illustrate differences in peptide accessibility and protectability across the states.

Supplementary references

1. Zheng, S. Q. *et al.* MotionCor2: anisotropic correction of beam-induced motion for improved cryo-electron microscopy. *Nat Methods* **14**, 331–332 (2017).
2. Rosenthal, P. B. & Henderson, R. Optimal determination of particle orientation, absolute hand, and contrast loss in single-particle electron cryomicroscopy. *J Mol Biol* **333**, 721–745 (2003).
3. Kimanius, D., Dong, L., Sharov, G., Nakane, T. & Scheres, S. H. W. New tools for automated cryo-EM single-particle analysis in RELION-4.0. *Biochem J* **478**, 4169–4185 (2021).
4. Jakobi, A. J., Wilmanns, M. & Sachse, C. Model-based local density sharpening of cryo-EM maps. *eLife* **6**, e27131 (2017).
5. Vagin, A. & Teplyakov, A. Molecular replacement with MOLREP. *Acta Crystallogr D Biol Crystallogr* **66**, 22–25 (2010).
6. Burnley, T., Palmer, C. M. & Winn, M. Recent developments in the CCP-EM software suite. *Acta Crystallogr D Struct Biol* **73**, 469–477 (2017).
7. Tamman, H. *et al.* Structure of SpoT reveals evolutionary tuning of catalysis via conformational constraint. *Nat Chem Biol* 1–12 (2022)
8. Kurata, T. *et al.* RelA-SpoT Homolog toxins pyrophosphorylate the CCA end of tRNA to inhibit protein synthesis. *Mol. Cell* (2021)
9. Horvatek, P. *et al.* Inducible expression of (pp)pGpp synthetases in Staphylococcus aureus is associated with activation of stress response genes. *PLOS Genetics* **16**, e1009282 (2020).
