# Supplementary material for: Deciphering the allosteric regulation of mycobacterial inosine-5′-monophosphate dehydrogenase
Source: Nat Commun. 2024 Aug 6;15:6673. doi: 10.1038/s41467-024-50933-6 (PMC11303537; doi:10.1038/s41467-024-50933-6)
Supplement: Supplementary file 3 — Description of Additional Supplementary Files [file 41467_2024_50933_MOESM3_ESM.pdf]

Description of Additional Supplementary Files

File Name: Supplementary Movie 1

Description: Overview of the integrated model for the allosteric regulation of mycobacterial IMPDH. The first part of the movie shows the regulation within the context of the purine biosynthetic pathway. The second part provides structural details of the molecular mechanism of the regulation.
