# Supplementary material for: Deciphering the allosteric regulation of mycobacterial inosine-5′-monophosphate dehydrogenase
Source: Nat Commun. 2024 Aug 6;15:6673. doi: 10.1038/s41467-024-50933-6 (PMC11303537; doi:10.1038/s41467-024-50933-6)
Supplement: Supplementary file 5 — Reporting Summary [file 41467_2024_50933_MOESM5_ESM.pdf]

nature portfolio

Corresponding author(s): T. Kouba, I. Pichová

Last updated by author(s): Jul 8, 2024

Reporting Summary

Nature Portfolio wishes to improve the reproducibility of the work that we publish. This form provides structure for consistency and transparency in reporting. For further information on Nature Portfolio policies, see our Editorial Policies and the Editorial Policy Checklist.

Statistics

For all statistical analyses, confirm that the following items are present in the figure legend, table legend, main text, or Methods section.

n/a	Confirmed	
☐	☒	The exact sample size (*n*) for each experimental group/condition, given as a discrete number and unit of measurement
☒	☐	A statement on whether measurements were taken from distinct samples or whether the same sample was measured repeatedly
☒	☐	The statistical test(s) used AND whether they are one- or two-sided<br>*Only common tests should be described solely by name; describe more complex techniques in the Methods section.*
☒	☐	A description of all covariates tested
☒	☐	A description of any assumptions or corrections, such as tests of normality and adjustment for multiple comparisons
☐	☒	A full description of the statistical parameters including central tendency (e.g. means) or other basic estimates (e.g. regression coefficient) AND variation (e.g. standard deviation) or associated estimates of uncertainty (e.g. confidence intervals)
☒	☐	For null hypothesis testing, the test statistic (e.g. *F*, *t*, *r*) with confidence intervals, effect sizes, degrees of freedom and *P* value noted<br>*Give P values as exact values whenever suitable.*
☒	☐	For Bayesian analysis, information on the choice of priors and Markov chain Monte Carlo settings
☒	☐	For hierarchical and complex designs, identification of the appropriate level for tests and full reporting of outcomes
☒	☐	Estimates of effect sizes (e.g. Cohen's *d*, Pearson's *r*), indicating how they were calculated

Our web collection on statistics for biologists contains articles on many of the points above.

Software and code

Policy information about availability of computer code

Data collection	All relevant information regarding the code used in this study is available at the respective GitHub depository: https://doi.org/10.5281/zenodo.11047778
Data analysis	All relevant information regarding the code used in this study is available at the respective GitHub depository: https://doi.org/10.5281/zenodo.11047778

For manuscripts utilizing custom algorithms or software that are central to the research but not yet described in published literature, software must be made available to editors and reviewers. We strongly encourage code deposition in a community repository (e.g. GitHub). See the Nature Portfolio guidelines for submitting code & software for further information.

Data

Policy information about availability of data

All manuscripts must include a data availability statement. This statement should provide the following information, where applicable:

- Accession codes, unique identifiers, or web links for publicly available datasets
- A description of any restrictions on data availability
- For clinical datasets or third party data, please ensure that the statement adheres to our policy

The atomic coordinates are deposited in the Research Collaboratory for Structural Bioinformatics Protein Data Bank (RCSB PDB) with accession codes: PDB 8PW3 (MsmGuaB2 apo), PDB 8Q65 (MsmGuaB2-ATP), PDB 8QQV (MsmGuaB2-ATP+IMP extended), PDB 8QQW (MsmGuaB2-ATP+IMP compressed), PDB 8QQX

(MsmGuaB2-ATP+IMP intermediate), PDB 8QQP (MsmGuaB2-ATP+GTP compressed), PDB 8QQQ (MsmGuaB2-ATP+GTP less compressed), PDB 8QQR (MsmGuaB2-ATP+ppGpp compressed), PDB 8QQT (MsmGuaB2 -ATP+ppGpp less compressed). The cryo-EM maps are deposited in the Electron Microscopy Data bank (EMDB) under accession codes: EMD–17988 (MsmGuaB2 apo), EMD–18184 (MsmGuaB2-ATP), EMD–18606 (MsmGuaB2-ATP+IMP extended), EMD–18607 (MsmGuaB2-ATP +IMP compressed), EMD–18608 (MsmGuaB2-ATP+IMP intermediate), EMD–18600 (MsmGuaB2-ATP+GTP compressed), EMD–18601 (MsmGuaB2-ATP+GTP less compressed), EMD–18602 (MsmGuaB2-ATP+ppGpp compressed), EMD–18604 (MsmGuaB2 -ATP+ppGpp less compressed).

Data on the SAXS experiments are deposited in the Small Angle Scattering Biological Data Bank (SASBDB) under the following accession codes: SASDUM5 (MsmGuaB2 apo), SASDUN5 (MsmGuaB2 IMP), SASDUP5 (MsmGuaB2 ATP), SASDUQ5 (MsmGuaB2 ATP+IMP) and SASDUR5 (MsmGuaB2 GTP).

The source data for all biochemical assays have been deposited in the Zenodo repository and are available at the following URL: https://doi.org/10.5281/zenodo.12646467

The HDX-MS datasets generated in this study have been deposited in the Zenodo repository and are available at the following URL: https://doi.org/10.5281/zenodo.12697518

Research involving human participants, their data, or biological material

Policy information about studies with human participants or human data. See also policy information about sex, gender (identity/presentation), and sexual orientation and race, ethnicity and racism.

Reporting on sex and gender	Not applicable
Reporting on race, ethnicity, or other socially relevant groupings	Not applicable
Population characteristics	Not applicable
Recruitment	Not applicable
Ethics oversight	Not applicable

Note that full information on the approval of the study protocol must also be provided in the manuscript.

Field-specific reporting

Please select the one below that is the best fit for your research. If you are not sure, read the appropriate sections before making your selection.

☒ Life sciences ☐ Behavioural & social sciences ☐ Ecological, evolutionary & environmental sciences

For a reference copy of the document with all sections, see nature.com/documents/nr-reporting-summary-flat.pdf

Life sciences study design

All studies must disclose on these points even when the disclosure is negative.

Sample size	Not applicable
Data exclusions	Not applicable
Replication	Not applicable
Randomization	Not applicable
Blinding	Not applicable

Reporting for specific materials, systems and methods

We require information from authors about some types of materials, experimental systems and methods used in many studies. Here, indicate whether each material, system or method listed is relevant to your study. If you are not sure if a list item applies to your research, read the appropriate section before selecting a response.

Materials & experimental systems

n/a	Involved in the study
☒	☐ Antibodies
☒	☐ Eukaryotic cell lines
☒	☐ Palaeontology and archaeology
☒	☐ Animals and other organisms
☒	☐ Clinical data
☒	☐ Dual use research of concern
☒	☐ Plants

Methods

n/a	Involved in the study
☒	☐ ChIP-seq
☒	☐ Flow cytometry
☒	☐ MRI-based neuroimaging

Plants

Seed stocks	Not applicable
Novel plant genotypes	Not applicable
Authentication	Not applicable
